# Psychological and psychosocial determinants of COVID‐related handwashing behaviours: A systematic review

**DOI:** 10.1002/cl2.1421

**Published:** 2024-07-15

**Authors:** Rachel Leonard, Sean R. O'Connor, Jennifer Hanratty, Ciara Keenan, Yuan Chi, Jenny Ferguson, Ariana Axiaq, Anna Volz, Ceri Welsh, Kerry Campbell, Victoria Hawkins, Sarah Miller, Declan Bradley, Martin Dempster

**Affiliations:** ^1^ School of Psychology Queen's University Belfast Belfast UK; ^2^ Centre for Evidence and Social Innovation Queen's University Belfast Belfast UK; ^3^ National Children's Bureau Belfast UK; ^4^ Yealth Network, Beijing Yealth Technology Co., Ltd. Shanghai China; ^5^ Centre for Effective Education Queen's University Belfast Belfast UK; ^6^ Centre for Public Health Queens University Belfast Belfast UK

**Keywords:** anxiety, COVID‐19, handwashing, social norms

## Abstract

**Background:**

The COVID‐19 pandemic, caused by the SARS‐CoV‐2 virus, has resulted in illness, deaths and societal disruption on a global scale. Societies have implemented various control measures to reduce transmission of the virus and mitigate its impact. Individual behavioural changes are crucial to the successful implementation of these measures. One commonly recommended measure to limit risk of infection is frequent handwashing. It is important to identify those factors that can predict the uptake and maintenance of handwashing.

**Objectives:**

We aimed to identify and synthesise the evidence on malleable psychological and psychosocial factors that determine uptake and adherence to handwashing aimed at reducing the risk of infection or transmission of COVID‐19.

**Search Methods:**

We searched various literature sources including electronic databases (Medline ALL, Child Development & Adolescent Studies, ERIC, PsycInfo, CINAHL and Web of Science), web searches, conference proceedings, government reports, other repositories of literature and grey literature. The search strategy was built around three concepts of interest including (1) context (terms relating to COVID‐19), (2) behaviour of interest and (3) terms related to psychological and psychosocial determinants of COVID Health‐Related Behaviours and adherence or compliance with handwashing, to capture malleable determines. Searches capture studies up until October 2021.

**Selection Criteria:**

Eligibility criteria included observational studies (both retrospective and prospective) and experimental studies that measure and report malleable psychological and psychosocial determinants and handwashing at an individual level, amongst the general public. Screening was supported by the Cochrane Crowd. Titles and abstracts were screened against the eligibility criteria by three independent screeners. Following this, all potentially relevant studies were screened at full‐text level by the research team. All conflicts between screeners were resolved by discussion between the core research team.

**Data Collection and Analysis:**

All data extraction was managed in EPPI‐Reviewer software. All eligible studies, identified through full‐text screening were extracted by one author. We extracted data on study information, population, determinant, behaviour and effects. A second author checked data extraction on 20% of all included papers. All conflicts were discussed by the two authors until consensus was reached.

We assessed methodological quality of all included studies using an adapted version of the Joanna Briggs Institute Quality appraisal tool for cross‐sectional studies.

**Main Results:**

Our initial searches yielded 23,587 results, of which 56 studies were included in this review. The included studies were cross sectional in design, came from 22 countries and had a combined sample of 199,376 participants. The vast majority of studies had samples from the general public, with eight of the studies focusing on specific samples. All included studies considered people over the age of 18. The quality of the majority of the studies was good (*n* = 30 rated low risk of bias), with 8 rated high risk of bias, predominately due to lack of reporting of recruitment, sample characteristics and methodology. Thirty‐four studies were included in the narrative synthesis and 28 in the meta‐analysis.

Findings indicated that emotions about COVID‐19 (worry [0.381, confidence interval [CI] = 0.270–0.482, *I* ^2^ = 92%) and anxiety (0.308, CI = 0.154–0.448, *I* ^2^ = 91%]), knowledge of COVID‐19 (0.323, CI = 0.223–0.417, *I* ^2^ = 94%), and perceived social norms (0.303, CI = 0.184–0.413, *I* ^2^ = 92%) were among the malleable determinants most associated with handwashing. Perceived severity (0.006, CI = ‐0.011–0.023) and susceptibility of COVID‐19 (0.041, CI = −0.034 to 0.115) had little to no effect on handwashing behaviour.

**Authors' Conclusions:**

Understanding the effects of various malleable determinants on COVID‐related handwashing can aid in the development and implementation of interventions and public health campaigns to promote handwashing behaviour in potential new waves of COVID‐19 or other respiratory infections. Emotions about COVID, knowledge of COVID and perceived social norms warrant further consideration in future research and policy.

## 1 PLAIN LANGUAGE SUMMARY

Knowledge, anixety, worry and social norms related to COVID affect handwashing.

### 1.1 What is this review about?

Health‐protective behaviours, such as handwashing, will be vital to reducing risk of infection and transmission in potential new waves of COVID. Therefore, it is important to understand the factors that influence this behaviour and that can be modified.

This review examined the modifiable psychological or psychosocial determinants of handwashing.

### 1.2 What is the aim of this review?

This Campbell systematic review examines the determinants of handwashing in studies conducted during the COVID‐19. The review summarizes evidence from 56 studies.

### 1.3 What are the main findings of this review?

#### 1.3.1 What studies are included?

This review included studies that looked at different determinants of handwashing. We included 56 studies in the review, 28 of these were included in our meta‐analysis, and 34 were described narratively. The studies were all conducted during the COVID‐19 pandemic and were conducted in 22 different countries. Many of the studies were good quality however 8 had some important weaknesses, including not providing enough detail about the sample, about how handwashing was measured and about the determinant they included.

#### 1.3.2 What determinants were associated with handwashing?

Determinants such as knowledge of COVID, worry and anxiety about COVID and social norms were the most associated with COVID‐related handwashing. Perceived susceptibility to COVID and perceived severity of COVID had little to no effect on handwashing.

### 1.4 What do the findings of this review mean?

Understanding what determinants affect handwashing behaviour can help us develop better public campaigns for potential future waves of COVID or other respiratory infections. Knowledge, social norms, emotions (such as anxiety and worry) should be the target of future interventions aiming to increase handwashing to minimise respiratory infections.

### 1.5 How up‐to‐date is this review?

The authors of this review employed search strategies intended to capture studies up until October 2021.

## 2 SUMMARY OF FINDINGS

**Summary of findings 1**

Summary of findings:

**Table:** 

Determinant	Effect size	95% CI	*Q*	*I* ^2^	*τ* ^2^	*k*
COVID‐related anxiety	*r* = 0.308***	0.154, 0.448	22.981	91%	0.019	3
COVID‐related worry	*r* = 0.381***	0.270, 0.482	35.762	92%	0.014	4
Perceived control	*r* = 0.185***	0.105, 0.262	7.013	57%	0.004	4
Attitudes	*r* = 0.264***	0.118, 0.399	84.743	94%	0.033	6
Self‐efficacy	*r* = 0.265***	0.146, 0.376	48.718	90%	0.021	6
Perceived effectiveness	*r* = 0.186***	0.090, 0.278	9.390	79%	0.006	3
Perceived risk	*r* = 0.202***	0.155, 0.248	2.237	0%	0	5
Perceived severity	*r* = 0.006	−0.011, 0.023	0.319	0%	0	3
Perceived susceptibility	*r* = 0.041	−0.034, 0.115	57.268	93%	0.006	5
Social norms	*r* = 0.291***	0.138, 0.431	34.509	91%	0.025	4
Knowledge about behaviour	*r* = 0.261*	0.007, 0.484	60.292	97%	0.051	3
Knowledge about the disease	*r* = 0.337***	0.238, 0.428	65.716	92%	0.016	6

Abbreviations: CI, confidence interval; *I* ^2^, percentage of variability due to between‐study heterogeneity; *k*, number of effect sizes; *Q*, test for heterogeneity; *r*, correlations; SMD, standardised mean difference; *τ* ^2^, random effects variance component.

*p < 0.05

***p < 0.001.

## 3 BACKGROUND

### 3.1 The problem, condition or issue

Severe acute respiratory coronavirus 2 (SARS‐CoV‐2) emerged in late 2019 and spread rapidly around the globe (Cucinotta & Vanelli, 2020; Wu et al., 2020). The pandemic of COVID‐19 disease, caused by SARS‐CoV‐2, has resulted in short and long‐term illness, deaths and societal disruption. Societies implemented control measures to reduce the transmission of the virus. Individual behaviour change is crucial to the success of these measures through reducing the frequency of social contacts, mitigating the risk of those social contacts and reducing the amount of time that infectious people are in contact with others whom they may infect. Despite vaccine programmes being introduced in December 2020, waning immunity and the evolution of new variants, indicate the significance of behavioural measures to reduce the spread (Girum et al., 2021; Michie & West, 2020).

The behaviours to reduce the risk of catching or spreading SARS‐CoV‐2 including: handwashing or use of hand sanitiser, wearing masks or face coverings, physical distancing, social distancing, isolation or quarantine, respiratory hygiene, cleaning surfaces, avoiding touching the ‘T‐zone’ (mouth, nose and eyes) (Elder et al., 2014) as well as other composite measures that include these behaviours.

The evidence for the effectiveness of these measures has been established during previous pandemics of similar serious viral respiratory infections such as pandemic Influenza A (H1N1), SARS and MERS (Flumignan et al., 2020; Jefferson et al., 2020; Seto et al., 2003; Warren‐Gash et al., 2013; Webster et al., 2020; West et al., 2020). It is important to synthesise the evidence on the determinants of these measures during the COVID‐19 pandemic, that may be applied to future pandemics of influenza and other serious respiratory infectious diseases.

### 3.2 Exposure/determinants

The exposure in this review was psychological or psychosocial determinants of handwashing. To be included, determinants were malleable factors that could, theoretically, be changed by a public health intervention.

### 3.3 Why it is important to do this review

Handwashing cannot be effective on a societal level if it is not adopted widely and consistently. Variables such as individual health beliefs, social support, culture, and social norms can all influence the likelihood of someone undertaking and maintaining health behaviours such as handwashing. To develop appropriate public health interventions to improve uptake and adherence to handwashing, including effective messaging, it is important to understand the malleable factors that influence this behaviour. We identified and examined all existing research evidence that described a relationship between any malleable factor or determinant (or those that can be most effectively targeted as part of public health interventions) and handwashing in the context of SARS‐CoV‐2.

In this review, we are interested in the evidence on malleable and non‐malleable psychological and psychosocial factors associated with uptake and adherence to health protective behaviours. Malleable determinants in this EGM refer to psychological and psychosocial factors that can be developed, shaped or altered. Factors such knowledge, access to information, emotions, and perceptions. Non‐malleable determinants in this EGM refer to factors or attributes that are fixed or unchangeable through public health intervention. Factors such as age, sex, income, past behaviour, and health status.

In any future severe viral outbreaks, health‐protective behaviours, such as handwashing, will be vital to reducing risk of infection and transmission. Non‐pharmaceutical interventions that are designed to improve the uptake and adherence to protective behaviours are essential in an outbreak, and in particular when vaccines and treatments are not yet established. The effectiveness of these behaviour change interventions will be determined, to some extent, by how they address the psychological and psychosocial variables that influence behaviour. To optimise public health intervention, we need to know which specific variables are most likely to influence the target behaviours, such as handwashing, in this context. Evidence gathered in the context of COVID‐19 can inform who, when and under what circumstances people do or do not adopt recommended preventive behaviours.

There are a number of related published and ongoing reviews on individual determinants of COVID‐19 health‐related behaviours but none with the broad scope of this review. Using robust search, retrieval, and methodological approaches to minimise potential sources of bias, this review examines the existing and emerging evidence on determinants of handwashing in the context of the COVID‐19 pandemic.

### 3.4 Overview of the COHeRe project

COHeRe is a UKRI funded project https://www.qub.ac.uk/schools/psy/Research/OurResearchThemes/HealthWelfareClinicalPsychology/COHeRe/ made up of a team with substantial expertise in systematic reviews, health behaviour and infectious diseases. The overall aim of the project was to identify, synthesis, and examine evidence on determinates of COVID‐19 health‐related behaviours. The specific behaviours of interest were as follows:

- Handwashing
- Wearing masks/face coverings
- Physical Distancing
- Social Distancing
- Isolation/quarantine
- Respiratory hygiene
- Cleaning surfaces
- Avoiding t‐zone
- Other composite measures that include the above.

During Phase 1 of the project a rapid review was conducted, which examined determinants of protective behaviours during COVID‐19 and during previous outbreaks of similar serious respiratory infections, for example, SARS, MERS and H1N1 (swine flu) (Hanratty et al., 2021). Of the 233 studies included in the rapid review, 54 were conducted in the context of COVID‐19, while the remainder were conducted in the context of other respiratory infections. Over the course of conducting the rapid review, it became apparent that the evidence base examining determinants in the context of COVID‐19 was rapidly expanding and further identification and examination was needed of this new evidence.

On this basis, further funding was secured to conduct Phase 2 of the project, which identified and mapped the existing evidence (published and unpublished between January 2020 and October 2021) on malleable and non‐malleable psychological and psychosocial factors that determine uptake and adherence to behaviours aimed at reducing the risk of infection or transmission of COVID‐19 (Hanratty et al., 2022, 2023). As of 1 June 2022 the Evidence and Gap Map (EGM) includes 1034 records https://eppi.ioe.ac.uk/eppi-vis/login/open?webdbid=188.

This current review is the final phase of the wider project. Based on those studies included in the EGM we further examined these, through a series of systematic reviews examining which malleable determinants (or those that can be most effectively targeted as part of public health interventions) are more closely associated with uptake and maintenance of individual protective behaviours. This current review examines the protective behaviour of handwashing, however is part of a series of reviews considering the 8 other behaviours of interest.

## 4 OBJECTIVES

We intended to identify and synthesise the existing evidence on malleable psychological and psychosocial factors that determine uptake and adherence to handwashing that can reduce the risk of infection or transmission of COVID‐19.

## 5 METHODS

None

### 5.1 Criteria for considering studies for this review

#### 5.1.1 Types of studies

This systematic review contains studies that quantify the relationship between a malleable determinant and handwashing. Included study designs consisted of observational studies (both retrospective and prospective) and experimental studies that measure and report malleable psychological and psychosocial determinants and handwashing at an individual level. We did not include narrative reviews, modelling studies, letters, editorials, opinion pieces, news, commentaries, or any other publications that did not report primary data.

#### 5.1.2 Types of participants

The population of interest is members of the general public, of any age. Within the group of studies of the general public, we included studies on specific groups of people that may be at increased risk of catching the virus for example, people who work in essential retail services. Similarly, we included studies of specific patient groups at increased risk of becoming seriously ill if infected, for example, those with existing chronic respiratory disorders. However, we did not include studies on health care workers (HCWs), defined as someone who works in a hospital or health care setting or delivers health care in the community. This population typically have, or should have additional knowledge, training and resources to support the adoption of behaviours to mitigate against the increased risk of exposure to infectious diseases. A rapid review on barriers and facilitators to HCWs adherence to infection prevention and control guidelines has been published (Houghton et al., 2020). For those studies that included both HCWs and the public, were only included if data on the public is presented separately from data on healthcare workers.

### 5.2 Exposure/determinants

The exposure in this review was psychological or psychosocial determinants of handwashing. To be included determinants were malleable factors that could, theoretically, be changed by a public health intervention.

We developed 10 categories of determinants for phase 2 of this project. These included, behaviour, cognition, demographics, disease, emotions, health status, information, intervention, knowledge and other Table 1. Each category was divided into subcategories of various determinants. As above, only malleable determinants were included in this review. Therefore, the following determinants were included:

**Table 1:** Determinant categories and subcategories.

**Determinant category**	**Subcategories**				
***Emotions***	Feelings about the disease	'Other’ emotion‐related determinants (e.g., general emotional state or mood)			
***Cognition*** (thoughts or perceptions)	About the protective behaviours	About COVID‐19	Motivations to engage in behaviour	Social cognition (e.g., perceived social norms)	Cognitive capacity indicating a person's ability to understand or retain information
***Knowledge*** (knowledge of)	Protective behaviours	COVID‐19	Any other types of assessed knowledge, such as knowledge of regulations or knowledge of vaccines		
***Information***	Seeking and consuming information	The quality or source of information	Public health messaging (e.g. message content or framing)		
***Other***	Beliefs (e.g., political beliefs)	social (e.g., social capital, social networks)	Practical resources (i.e., access to masks)	Cultural (i.e. collectivist vs. individualist cultures)	

**Emotions** captured determinants related to feelings about the disease and ‘other’ emotion‐related determinants for example general emotional state or mood.

**Cognition** was broken down into six subcategories: thoughts or perceptions about the protective behaviours; about COVID‐19; motivations; social cognition (e.g., perceived social norms); cognitive capacity indicating a person's ability to understand or retain information; ‘other’ to capture any other cognitive determinant that did not fit into the previous five subcategories.

**Knowledge** included determinants relating to knowledge about protective behaviours, knowledge about the disease and any other types of assessed knowledge, such as knowledge of regulations or knowledge of vaccines.

**Information** included seeking and consuming information, the quality or source of information, and determinants related to public health messaging, for example, message content or framing.

**Other** was the final category of determinants and includes any determinants that did not fit within the previous broad categories. This was divided into subcategories of beliefs, for example, political beliefs, social (e.g., social capital, social networks), practical resources such as access to masks, paid sick leave, time included time since the outbreak began, cultural determinants such as collectivist vs individualist cultures, and a final ‘other’ subcategory for any remaining determinant that did not fit into the previous subcategories.

The determinants of behaviour, demographics, disease, and health status were not included as these were categorised as non‐malleable. We also did not include studies that examined interventions as a determinant of handwashing as this will be analysed in a separate review.

Comparators were the absence of the determinant (compared to its presence) or, where a determinant is presented as a continuous measure, then analysis will be based on correlation between handwashing and determinants.

We included studies that measured determinants at an individual level and group level, for example, country‐level data on the number of cases.

We included studies on self‐reported or observed determinants. Self‐reports included actual or perceived determinants, for example ‘risk of contracting the virus’ could be measured by quantifying actual risk based on individual circumstances and behaviour or through self‐reported perceived risk.

#### 5.2.1 Types of outcome measures

While our searches sought to identify evidence on commonly recommended behaviours to mitigate human‐to‐human spread of COVID‐19 as described by (West et al., 2020), this current review focuses on handwashing only. We define handwashing as, washing hands more frequently with soap and water or the use of hand sanitizer if handwashing facilities are not available.

We included studies on actual handwashing behaviour, through self/other report and/or observation, measured at the individual level. We excluded studies that measured intended behaviour or hypothetical behaviour.

##### Primary outcomes

The primary outcome of this review was handwashing. No secondary outcome was considered.

### 5.3 Search methods for identification of studies

To ensure that the literature contained in the review was relevant and useful to key stakeholders, it was important that the literature retrieval methods followed high‐quality standards and all searches were conducted and reported following Campbell Collaboration guidelines (White et al., 2020).

Information retrieval specialist author (CK) developed and piloted a search strategy with input from clinical and behaviour change expert authors (DB and MD). This strategy was further refined by CK following expert advice from a Campbell information retrieval specialist during the editorial/peer review of the protocol. Searches strageries in the current review capture studies up until October 2021.

The search strategy was built around three concepts of interest;

(1) Context (terms relating to COVID‐19). For concept one, we used an innovative and tested COVID‐19 search strategy was developed for use by NICE information specialists and was updated as recently as 21 June 2021 (Levay & Finnegan, 2021). An example of the search string was piloted in Medline (Ovid) and is presented in Table 3.
(2) Behaviours of interest.
(3) Terms related to psychological and psychosocial determinants of COVID Health‐Related Behaviours and adherence or compliance with recommended behaviours, to capture both malleable and non‐malleable determinants.

For concept 2 and 3 the terms used were based on those used in the rapid review (Hanratty et al., 2021) which itself was informed through consultation with the Behaviour Change Group formed in response to COVID‐19 by the Public Health Agency, Northern Ireland. The terms were then piloted and refined in two databases, with unique terms added and redundant or duplicate terms removed (Table 2).

**Table 2:** Medline (Ovid) search strategy.

Ovid MEDLINE(R) ALL <1946 to 3 September 2021>		
1	SARS‐CoV‐2/or COVID‐19/	103,591
2	(corona* adj1 (virus* or viral*)).ti,ab.	2364
3	(CoV not (Coefficien* or ‘co‐efficien*’ or covalent* or Covington* or covariant* or covarianc* or ‘cut‐off value*’ or ‘cutoff value*’ or ‘cut‐off volume*’ or ‘cutoff volume*’ or ‘combined optimi?ation value*’ or ‘central vessel trunk*’ or CoVR or CoVS)).ti,ab.	51,911
4	(coronavirus* or 2019nCoV* or 19nCoV* or ‘2019 novel*’ or Ncov* or ‘n‐cov’ or ‘SARS‐CoV−2*’ or ‘SARSCoV‐2*’ or SARSCoV2* or ‘SARS‐CoV2*’ or ‘severe acute respiratory syndrome*’ or COVID*2).ti,ab.	181,470
5	or/1‐4	187,096
6	limit 5 to yr = ‘2020‐Current’	173,962
7	(6 and english.lg.) not (letter or historical article or comment or editorial or news).pt. not (Animals/not humans/)	134,173
8	(Mask or masks or face?mask* or Face cover*).ti,ab.	42,975
9	(face adj2 (shield or shields)).ti,ab.	414
10	(((Hand or hands) adj2 hygiene) or Handwash* or (Wash* adj2 hand*)).ti,ab.	11,132
11	(hand adj1 clean*).ti,ab.	256
12	(hand adj2 saniti*).ti,ab.	683
13	(hand adj2 disinfect*).ti,ab.	783
14	Respiratory hygiene.ti,ab.	79
15	Respiratory etiquette.ti,ab.	27
16	((cough* or sneeze*) and (sleeve or arm or elbow or tissue or etiquette)).ti,ab.	2752
17	(tissue and (dispose or disposal or bin or hygiene)).ti,ab.	3414
18	universal hygiene.ti,ab.	10
19	Social Isolation/or Patient Isolation/	19,284
20	(self‐isolate or self‐isolation or self‐isolating).ti,ab.	724
21	(mass adj2 (behav* or gather*)).ti,ab.	1690
22	(social distance or social distancing).ti,ab.	6625
23	stay at home.ti,ab.	1465
24	stay home.ti,ab.	314
25	((work* adj2 home) or telecommute or telework* or (remote* adj2 work*)).ti,ab.	5262
26	(Physical adj2 distanc*).ti,ab.	2595
27	(touch* and (mouth or mouths or face or faces or nose or noses or t‐zone)).ti,ab.	1635
28	disinfect*.ti,ab.	31,760
29	lockdown.ti,ab.	8167
30	quarantine.ti,ab.	7821
31	(nonpharmaceutical or non‐pharmaceutical).ti,ab.	1831
32	(school closure or close school* or school closing).ti,ab.	389
33	or/8‐32	140,404
34	limit 33 to yr = ‘2020‐Current’	34,955
35	(34 and english.lg.) not (letter or historical article or comment or editorial or news).pt. not (Animals/not humans/)	31,455
36	7 and 35	20,298
37	exp Knowledge/	12,323
38	exp Health knowledge, Attitudes, Practice/	119,567
39	(Knowledg* or Personal* or Attitude* or Practice* or Habit* or belie* or Behav* or Need* or prevent* or Compliance or comply* or complied or Perception* or Protect* or Predict* or view* or barrier* or facilitator* or readiness or prepar* or ability* or insight or proficien* or procedur* or adher*).ti,ab.	10,617,318
40	or/37‐39	10,635,825
41	7 and 35 and 40	14,859

#### 5.3.1 Electronic databases

Based on the Queens's University Belfast database subscriptions, we searched the following key information sources to locate relevant primary research:

- Medline ALL (Ovid)
- Child Development & Adolescent Studies (EBSCOhost)
- ERIC (EBSCOhost)
- PsycInfo 1806‐present (OVID)
- CINAHL Plus (EBSCOhost)
- Web of Science Core Collection (the QUB subscription includes SCI‐expanded, SSCI, A&HCI, CPCI‐S, CPCI‐SSH, ESHI)

To locate relevant secondary research for inclusion in the EGM, we searched the following information resources:

- The Social Care Institute for Excellence (SCIE)
- The Cochrane Library
- Epistemonikos Covid‐19 evidence platform
- Norwegian Institute of Public Health living maps
- EPPI – centre
- COVID‐END

#### 5.3.2 Other sources

We searched for Grey literature across multiple sources. Grey literature is that which is not published, not peer reviewed, and not easily accessible. Sources of grey literature are varied and include government reports, privately and publicly funded research, conference proceedings, working papers, and posters. Some grey literature sources are captured in the Web of Science search, these include:

- Conference Proceedings Citation Index‐ Science (CPCI‐S)—1990‐present
- Conference Proceedings Citation Index‐ Social Science & Humanities (CPCI‐SSH)—1990‐present

We attempted to locate additional grey literature by searching sources such as the following:

- Google Scholar (We will search https://scholar.google.com/ using an incognito browser and the following strategy: (coronavirus| ‘2019 nCoV’| ‘2019 novel’| ‘2019 nCoV’| ‘2019 nCoV’| CoV |‘COVID 19’ |COVID19| ‘COVID 19’| ncov |‘SARS CoV2’| ‘SARS CoV 2’|‘severe acute respiratory syndrome Coronavirus 2’) (Psychological|Psychosocial)(behavior|behaviour) we will limit returns by ‘Since 2020’ filter and sort remaining records by relevance. We downloaded the first 1000 articles (which is the upper limit set by Google) using Harzing's Publish or Perish software.
- clinicaltrials.gov
- ISRCTN Registry (https://www.isrctn.com/)
- WHO International Clinical Trials Registry Platform (ICTRP) (https://www.who.int/clinical-trials-registry-platform/the-ictrp-search-portal)
  And by contacting and reviewing the information of the following key organisations in the UK with proven experience on the topics related to this project:
- King's Fund (https://www.kingsfund.org.uk/)
- National Institute for Health Research (https://www.nihr.ac.uk/)
- NHS Evidence (https://www.evidence.nhs.uk/)

We considered searching ProQuest dissertations and theses, however, we assessed that it was unlikely that any relevant doctoral theses would be complete and available in the timeframe of the virus.

We conducted a search of reference lists of previous reviews and eligible articles to identify any additional studies not identified through the electronic search. Finally, when we compiled a list of included studies, we contacted key experts in the field via email (categorised as ‘key’ if they have published five or more included studies) to ask whether they were aware of any unpublished or ongoing research that might not have been easily accessible to the research team.

To locate additional relevant grey literature for inclusion in the EGM, we searched for ongoing or unpublished reviews via:

- PROSPERO,
- Figshare and the
- Open Science Framework (OSF).

Any ongoing reviews were checked again before completion of the project, and, if still unpublished were excluded from the map.

#### 5.3.3 Search limits

Due to the limited language skills of the review team, we only included studies published in English.

We limited our search to exclude opinion pieces, letters, editorials and unpublished reports in databases where these limits are supported (See Table 3: lines 7 and 35). We did not use database limiters for studies on humans only as we found these limiters excluded a substantial number of potentially relevant papers not indexed as ‘human’ studies. Instead, we have opted to use an adaptation of the Cochrane search filter for human studies (line 7 and 35).

**Table 3:** Demographics of included studies.

Study	Country	Study design	Describe population	Age	Sex ‐number of women/girls	Sex ‐number of men/boys	Overall quality rating
Al‐Sejari (2021)	Kuwait	Cross sectional	General public (*n* = 1413)	Ranged from 18 to 99 years (mean = 39)	69.2% of sample	30.4% of sample	Low risk of bias
Al‐Shammary (2021)	Saudi Arabia	Cross sectional	General public (*n* = 400)	Mean 37.6 (10.8)	203	197	Low risk of bias
Al‐Wutayd (2021)	Saudi Arabia	Cross sectional	General public (*n* = 1323)	30–39: (27%)	645 (49%) female	678 (51%) were males,	Unclear risk of bias
Apanga (2021)	Ghana	Cross sectional	Pregnant women (*n* = 527)	*M* 26 (SD 5.9)	100%	0	Low risk of bias
Barrett (2021)	UK	Cross sectional	UK university students (*n* = 293)	73.4% 18–25 years; 26.6% over 25	189	100	Low risk of bias
Bogg (2020)	USA	Cross sectional	General public (*n* = 500)	*M* 45.4, SD 15.78	257	243	Low risk of bias
**Bruine de Bruin et al. (** **2020** **)**	USA	Cross sectional	General public (*n* = 6684)	Adults 20% 65 or over	3458 52%	48% 3226	Low risk of bias
Bruine de Bruin et al. (2020)	US	Cross sectional	General public (*n* = 5517)	20% were aged 65 and older	not reported	48% male	High risk of bias
Callaghan (2021)	USA	Cross sectional	General public (*n* = 5009)	Not reported	not reported	Not reported	Low risk of bias
Cowling et al. (2020)	Hong Kong	Cross sectional	General public (*n* = 12,965)	18–24: 1371 (13%) 25–34: 1210 (12%) 35–44: 1915 (19%)	61% 6308	39% 4026	Low risk of bias
Dixon et al. (2022)	United Kingdom ‐ Scotland	Cross sectional	General public (*n* = 2969)	16–24 years old: 273 25–34 years old: 385 35–44 years old: 360 45–54 years old: 540 55–64 years old: 607 65+ years old: 804	1,765 59.6%	1198 40.4%	Low risk of bias
Dwipayanti (2021)	Indonesia	Cross sectional	General public (*n* = 896)	*M* 35	543 (60.60%)	353 (39.40%)	Unclear risk of bias
Fujii (2021)	Italy, Japan, Korea, USA, China, UK	Cross sectional	General public (*n* = 5945)		3045 women	2900 men	Unclear risk of bias
Graupensperger (2021)	United States	Cross sectional	Young adult (*n* = 539) (recruited from university)	19.5 years (SD = 0.8)	58.8% women	Not reported	High risk of bias
Haliwa et al. (2020)	USA	Cross sectional	General public (*n* = 353)	Mean = 41.47 years, SD = 12.49, range: 19–84	59.8% women	Not reported	Low risk of bias
Hsing (2021)	USA	Cross sectional	General public (*n* = 71,851) (From US, Mexico, Hong Kong and Taiwan)	18–24 years: *n* (%) US 110 (3.6) Mexico 507 (12.9) Hong Kong 83 (7.0) Taiwan 4969 (7.8)	US 1683 (55.0) Mexico 2031 (51.6) Hong Kong 602 (50.4) Taiwan 31,407 (49.6)	US 1351 (44.2) Mexico 1867 (47.4) Hong Kong 562 (47.1) Taiwan 30,034 (47.4)	Unclear risk of bias
Iqbal (2021)	Pakistan	Cross sectional	General public (*n* = 1789)	18–25 (49.52%) 26–30 (27%) 31–40 (19.01%) >40 years: (4.47%)	949 (53.05%)	840 (46.95%)	High risk of bias
Jang et al. (2020)	Korea	Cross sectional	General public (*n* = 1,004)	19–29: (17.8%)	50.2%	49.8%	Low risk of bias
Jimenez (2020)	USA	Cross sectional	General public (*n* = 290)	*M* 37.12 (SD 12.03)	122 (40%)	180 (59%)	Low risk of bias
Jovančević & Milićević (2020)	Serbia/Latin‐America	Cross sectional	General public (*n* = 412)	Serbia: (M = 30.34; SD = 9.89) Latin America (*M* = 33.51, SD = 11.23)	Serbia (Female = 250) Latin‐America (Female = 95)	Serbia (*N* = 292, Male = 42) Latin‐America (*N* = 120, Male = 25)	Unclear risk of bias
Kebede (2020)	Ethiopa	Cross sectional	Visitors to a medical centre (*n* = 247)	*M* 30.5 (SD 10.2)	23% 58	77% 189	Unclear risk of bias
Kowalski (2020a) **Study 1**	Poland	Cross sectional	General public (*n* = 507)	44.07 (±14.41)	253 (49.9%)	Not reported	Low risk of bias
Kowalski (2020b) **Study 2**	Poland	Cross sectional	General public (*n* = 840)	29.94 (±10.39)	607 (72.3%)	Not reported	Low risk of bias
Lahiri (2021)	India	Cross sectional	General public (*n* = 2646)	aged ≤35 years (43.08%),	998 (37.72%)	1648 (62.28%)	High risk of bias
Lao et al. (2023)	China	Cross sectional	Residents in Hubei province (*n* = 229)	*M*(SD) 25.37(8.34)	141 (61.6%)	88 (38.4%)	Low risk of bias
Lee (2020)	South Korea, Ethiopia, and Democratic Republic of Congo	Cross sectional	General public (*n* = 748) (from South Korea, Ethiopia, and Democratic Republic of Congo)	South Korea *M* = 22.9, SD = 4.7 Ethiopia *M* = 26, SD = 5.7 Democratic Republic of Congo *M* = 26.1, SD = 4.2	South Korea female: 215(58.1%) Ethiopia female: 60 (34.3%) Democratic Republic of Congo female: 103(52.6%)	South Korea male: 155(42.9%) Ethiopia male: 115(65.7%) Democratic Republic of Congo male: 93(47.4%)	High risk of bias
Lee et al. (2021)	South Korea	Cross sectional	General public (*n* = 970)	*M* = 47.44 SD = 14.78	499 (51.4%)	471 (48.6%)	Unclear risk of bias
Li (2021)	USA	Cross sectional	Student pharmacists (*n* = 326)	18–26: 258 (79.1%) 27: 68 (20.9%)	242 females (74.2%)	84 males (25.8%)	Unclear risk of bias
Matkovic et al. (2021)	USA	Intervention study	General public (*n* = 344)	*M* = 32.69, SD = 11.60	54.1%	43.9%	Unclear risk of bias
Milman (2020)	USA	Cross sectional	General public (*n* = 408)	*M* 37.24 (SD 10.90)	174	233	Unclear risk of bias
Mousavi et al. (2022)	Afghanistan	Cross sectional	General pubic (*n* = 450)	17–26 (65.8%) 27–36 (22.9%) 37–46 (8.2%) 47–56 (2.2%) >57 (0.9%)	138 (28.4%)	322 (71.6%)	Unclear risk of bias
Nelson (2021)	USA	Cross sectional	Employees of Colorado State University (*n* = 508)	*M* 41.1 (SD12.5)	305 (60.0%)	200 (39.4%)	Unclear risk of bias
Norman (2020)	UK	Cross sectional	General public (*n* = 477)	*M* 46.22 (SD 15.20)	243 (50.9%)	234 (49.1%)	Low risk of bias
Owhonda (2022)	Nigeria	Cross sectional	General public (*n* = 1294)	*M* 39.6 (SD 11.9)	584 (45.1%)	710 (54.9%)	Unclear risk of bias
Ozdemir et al. (2022)	Singapore	Cross sectional	General public (*n* = 897)	*M* 42 (SD 12.8)	427 (47.6%)	470 (52.4%)	Low risk of bias
Pal et al. (2020)	India	Cross sectional	People with T1 Diabetes (*n* = 212)	*M* 25.1 (SD 4.3)	52% (111)	48% (101)	Unclear risk of bias
Pan (2020)	China	Cross sectional	Factory workers (*n *=* *3035)	<30 years of age (1552, 51.1%)	1423 (46.9%)	1612 (53.1%)	Low risk of bias
Prete (2020)	Italy	Cross sectional	General public (*n* = 618)	*M* 38.55 (SD = 15.26)	441 (71.4%)	177 (28.6%)	Unclear risk of bias
Qian (2020)	China	Cross sectional	General public (*n* = 1011) (from Wuhan % Shanghai)	Wuhan: 18–24 89 (21.6%) Shanghai: 18–24 75 (13.9%)	Not reported	Wuhan: 255 (50.0%) Shanghai: 255 (48.7)	Low risk of bias
Rattay (2021)	Germany	Cross sectional	General public (*n* = 27,957)	18–29: 19.1% 30–45: 30.1% 46–60: 28% >60: 22.8%	51% women *n* = 14,133	49% men *n* = 13,824	Unclear risk of bias
Rui (2021) **Study 1**	China	Cross sectional	General public (*n* = 321)	18–30: 55 (17.1% 31–45: 97 (30.2%) >46: 169 (52.6%)	167 (52%)	154 (48%)	Low risk of bias
Rui (2021) **Study 2**	China	Cross sectional	General public (*n* = 319)	18–30: 64 (20.1%) 31–45: 82 (25.7%) >46:173 (54.2%)	155 (48.6%)	164 (51.4)	Low risk of bias
Rui (2021) **Study 3**	China	Cross sectional	General public (*n* = 315)	18–30: 63 (20%) 31–45: 87 (27.6%) >46: 165 (52.4%)	174 (55.2%)	141 (44.8)	
Rui (2021) **Study 4**	China	Cross sectional	General public (*n* = 343)	18–30: 84 (24.5%) 31–45: 110 (32.1%) >46: 149 (43.4%)	186 (54.2%)	157 (45.8)	Low risk of bias
Rui (2021) **Study 5**	China	Cross sectional	General public (*n* = 329)	18–30: 74 (22.5%) 31–45: 86 (26.1%) >46: 169 (51.4%)	176 (53.5%)	153 (46.5)	Low risk of bias
Rui (2021) **Study 6**	China	Cross sectional	General public (*n* = 315)	18–30: 60 (19%) 31–45: 80 (25.4%) >46: 175 (55.6%)	152 (48.3%	163 (51.7)	Low risk of bias
Sengeh (2020)	Sierra Leone	Cross sectional	General public (*n* = 1,253)	18–39 (58%)	604 (48%)	648 (52%)	High risk of bias
**Sharma**	USA	Cross sectional	University students (*n* = 713)	*M* 24.61 (SD 8.60)	501 (70.3%)	176 (24.7%)	Unclear risk of bias
Shook (2020)	USA	Cross sectional	General public (*n* = 1,023)	*M* 46.32 (SD 16.57)	514 (50.8%)	497 (49.1%)	Unclear risk of bias
Si et al. (2021)	US	Cross sectional	General public (n = 1,019)	*M* 46.33 (SD 16.57)	514 female	497 male	Low risk of bias
Souliotis (2021)	Greece	Cross sectional	General public (*n* = 923)	25–54 (51.4%)	49.2%	50.8%	Unclear risk of bias
Stojanovic (2021)	Italy	Cross sectional	General public (*n* = 1332)	<25: (57%) 26–50 (33.3%) >51 (9.7%)	899 (68%)	422 (32%)	Low risk of bias
Trifiletti (2021)	Italy	Cross sectional	General public (*n* = 248)	*M* 34.78 (SD 14.76)	176 females	72 males	Unclear risk of bias
van den Broek‐Altenburg (2021)	Netherlands and Belgium	Cross sectional	General public The Netherlands (*n* = 2637) General public Flanders (*n* = 1678)	18–25: 483 (11.19%)	2250 (52.14%)	not reported	Low risk of bias
Wang (2021)	China	Cross sectional	Pregnant women (n = 15,428)	<30 (59.9%)	100%	None	Low risk of bias
Zewude et al. (2021)	Ethiopia	Cross sectional	People from the urban‐based informal economy (*n* = 384)	*M* 24.6 (SD 6.87)	161 (42.5%)	218 (57.5%)	Low risk of bias

We included only those studies which were conducted during the ongoing COVID‐19 pandemic. We included studies from Jan 2020 until the date of the final search.

### 5.4 Data collection and analysis

#### 5.4.1 Selection of studies

All search results were first screened on titles and abstracts against the eligibility criteria by three independent screeners. Screening at this first stage was supported by the Cochrane Crowd. We retrieved a full‐text copy of all potentially relevant studies during the title and abstract screening. Following this, all potentially relevant studies were screened independently by at least two reviewers from the research team at full‐text level. All conflicts between screeners were resolved by discussion between the core research team.

#### 5.4.2 Data extraction and management

All data extraction was managed in EPPI‐Reviewer software. All eligible studies, identified through full‐text screening were extracted by one author, who also completed the quality appraisal assessment. Any studies identified as ineligible during data extraction stage were listed as ‘excluded’. A second author checked the data extraction and risk of bias assessments on 20% of all included papers. The two people who completed the data extraction for each study discussed any discrepancies until they reach a consensus or, referred to a third author to make a final decision. In addition, the research team met on a weekly basis to discuss extraction and discrepancies, in aid coherence to the extraction protocol. Where data was not available or was missing within an included study, the research team attempted to obtain or clarify data from the relevant authors.

Extracted information included (Supporting Information 5):

- **Study information:** Author, year, country, study design, when the study was conducted, sample size.
- Population: description of the population, age, sex.
- **Exposure:** determinant measured, description of the determinant, who measured the determinant, type of measurement (observation, self‐reported, etc.), direction and quality of the scale.
- **Outcome**: behaviour measured, description of the behaviour, who measured the behaviour, type of measurement (observation, self‐reported, etc.), direction and quality of the scale.
- **Effects**: Narrative description of the finding, effect size information or sufficient numerical data to allow us to calculate the effect size.

#### 5.4.3 Quality appraisal

The JBI tool for cross‐sectional studies was used to assess the quality of included studies (The Joanna Briggs Institute, 2017; The Joanna Briggs Institute, 2020). After piloting the JBI tool on some known studies we decided to modify the tool to ensure that they are fit for our purposes (Supporting Information 6). We changed the wording of the second item ‘were the study subjects and the setting described in detail’ to ‘was the sample included in the study representative of the population of interest?’ to assess whether or not the sample was representative of the population of interest. We also changed the wording slightly, replacing condition and exposure with behaviours of interest and determinants, respectively.

The eight questions were answered with either ‘yes’, ‘no’, or ‘unclear’. For the questions on scale validity and reliability, we indicated whether a single‐item or multiple‐item scale was used and whether or not this was reliable and valid. Each study was rated either low, high or unclear risk of bias through adding up the total number of items answered ‘yes’. For example, >70% yes = Low Risk of Bias, 50%–70% yes = Unclear Risk of Bias, and <50% ‘Yes’ = High Risk of Bias.

#### 5.4.4 Measures of treatment effect

We extracted data on the relationship between handwashing and determinants of that behaviour. Outcomes were reported in both dichotomous and continuous data. The meta‐analysis was performed using Comprehensive Meta‐Analysis Version 4 (Comprehensive Meta‐Analysis Version 4, 2022), and conducted using correlation coefficients (*r*), as that was the effect size statistic most commonly reported in the papers. Therefore, data was extracted that allowed us to convert or calculate r. For example, where summary statistics were not presented, we extracted data such as means and standard deviations that allowed us to calculate a standardised mean difference that was then converted to r. Effect sizes were interpreted according to thresholds suggested by Cohen 1988: weak (*r* = 0.1), moderate (*r* = 0.3), and strong (*r* = 0.5).

#### 5.4.5 Unit of analysis issues

There are two reports that include multiple studies (Rui et al., 2021; Kowalski 2020a). Given that these separate studies utilised different samples, we treated as individual studies. Each individual study is referred to Author study 1, Author Study 2 and so on.

#### 5.4.6 Assessment of heterogeneity

Heterogeneity was assessed first, through visual inspection of the forest plot and checking for overlap of confidence intervals and second through the *Q*, *I* ^2^ and *τ* ^2^ statistic. Investigation of the source of heterogeneity is addressed in data synthesis section.

#### 5.4.7 Data synthesis

Given the diverse range of behaviour and determinant relationship examined across the included studies, we used random effects models, using inverse‐variance estimation. We conducted separate meta‐analyses for each determinant of the behaviour of interest, handwashing.

– Determinants were grouped based on previous mapping (Hanratty et al., 2023);
– Determinant groups were included in the meta‐analysis if they included data that was suitable for meta‐analysis (i.e. unadjusted data) and there was a minimum of three data points;
– We excluded adjusted estimates from meta‐analyses as there is considerable variation in the covariates used to adjust these estimates across studies and, therefore, we judged that the adjusted estimates were not suitable for statistical aggregation;
– Data that was not suitable was synthesised narratively.

##### Treatment of qualitative research

The review does not include qualitative research.

## 6 RESULTS

### 6.1 Description of studies

#### 6.1.1 Results of the search

**Figure 1:**
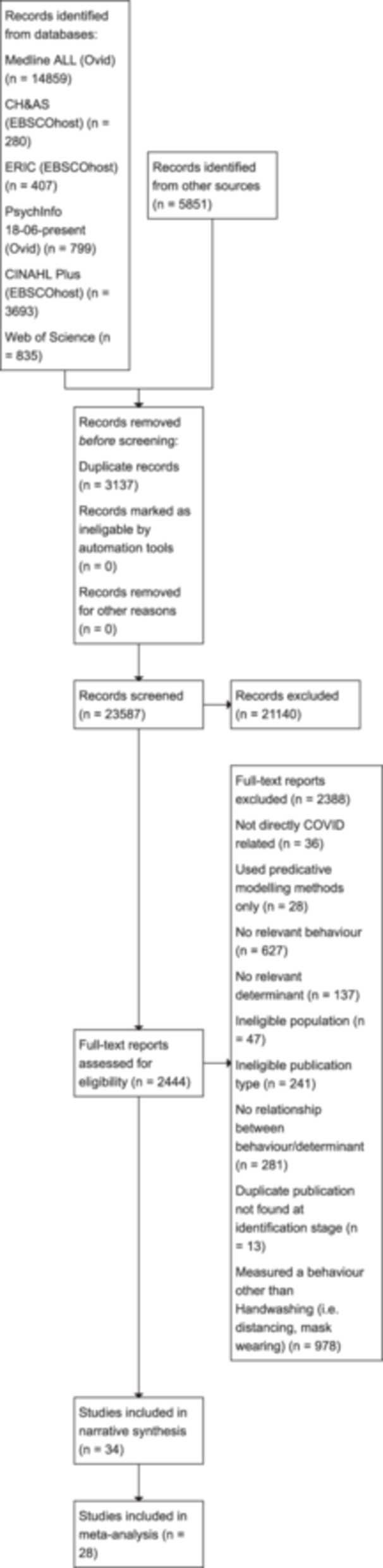
PRISMA flow diagram.

As seen in Figure 1, our searches yielded a total of 23,587 results. After screening out titles/abstracts we were left with 2444 results. Of these 2444 reports 2388 were excluded. Reasons included being directly COVID‐related, using predictive modelling methods, not relevant behaviour (including behaviours like mask‐wearing and distancing included in our other reviews reported elsewhere) or determinant, ineligible population or publication, no relationship measured between behaviour and determinant or a duplicate not found at the initial screening stage. Following full‐text screening of these results yielded 56 eligible studies.

#### 6.1.2 Included studies

A total of 56 studies were included in this review. Of these 56 studies, all used a cross sectional design. As detailed above, two of the reports (Kowalski 2020a (Kowalski et al., 2020a); Rui et al., 2021 (Rui et al., 2021) included separate studies. Kowalski 2020a reported two studies, and Rui et al., 2021 reported six separate studies. The 56 studies came from 22 different countries, with the majority coming from the USA (12). Other countries included China (10), UK (3), Italy (2) and Saudi Arabia (2). Four studies had data from across multiple countries (Fujii 2021 (Fujii et al., 2021); Jovančević & Milićević 2020 (Jovančević & Milićević, 2020); Lee 2020 (Lee et al., 2020); van den Broek‐Altenburg (van den Broek‐Altenburg & Atherly, 2021). Full details of included studies can be found in Table 3.

There was a total of 199,376 participants across the 56 studies, ranging from 71,851 (Hsing 2021 (Hsing Julianna et al., 2021) to 212 (Pal et al. 2020 (Pal et al., 2020). The vast majority of studies had samples from the general public, with eight of the studies focusing on specific samples. These included; pregnant women (Wang 2021 (Wang et al., 2021); Apanga 2021 (Apanga & Kumbeni, 2021), factory workers (Pan 2020 (Pan et al., 2020), people with type 1 diabetes (Pal et al., 2020), student pharmacists (Li 2021 (Li et al., 2021), visitors to a medical centre (Kebede 2020 (Kebede et al., 2020), and university students (Graupensperger 2021 (Graupensperger et al., 2021); Barrett 2021 (Barrett & Cheung, 2021).

All studies included participants over 18 years old. Reporting of age varied between studies, some providing mean age of participants, others providing percentage of age ranges and some not reporting age (Callaghan 2021 (Callaghan et al., 2021); Fujii 2021). For those studies that did report on age of participants, the average age was 35.5 years.

**Reported outcome:** Studies varied in their approaches to measuring hand washing. Measures ranged from scales (e.g. Hsing 2021) to single items (e.g., Are you regularly washing you hands with soap and water?, Pal et al., 2020). Handwashing was defined as using soap and water or using hand sanitizer within the included studies. Some studies measured adherence to specific hand washing guidance within the country of origin (e.g., Al‐Shammary 2021 (Al‐Shammary et al., 2021), some measured the frequency of handwashing (e.g., washed hands with soap or used hand sanitizer several times a day, Bruine de Bruin et al. 2020 (Bruine de Bruin et al., 2020), or measured handwashing following various activities (e.g. I have washed my hands every time I came into contact with objects or external environments, Trifiletti 2021 (Trifiletti et al., 2021).

**Determinants:** There were 18 determinants analysed across the 56 studies, including worry, perceived risk, knowledge, perceived barriers, and beliefs and motivation. Multiple determinants were reported within individual studies, for example Rui et al., 2021 reported on perceived self‐efficacy, perceived risk, perceived susceptibility, and knowledge. The most commonly reported determinant was perceived susceptibility of COVID‐19 (*n* = 25), followed by perceived severity of COVID‐19 (*n* = 21). Perceived effectiveness of handwashing (*n* = 3), fear of COVID (*n* = 3) and COVID‐related anxiety (*n* = 3) were the least reported determinants.

Following assessment of the data, 28 studies were deemed suitable to include in the meta‐analysis. These 28 studies reported on 12 determinants. A total of 34 studies were included in the narrative synthesis, reporting six determinants. Studies were considered not suitable for meta‐analysis due to not reporting unadjusted data. Given the multiple determinants reported in individual studies, 6 studies were included in both the narrative synthesis and meta‐analysis (Al‐Sejari 2021 (Al‐Sejari Maha and Al‐Ma'Seb Hend, 2021); Al‐Shammary 2021; Apanga 2021; Barrett 2021; Kowalski 2020b (Kowalski et al., 2020b); Norman 2020 (Norman et al., 2020).

#### 6.1.3 Excluded studies

A total of 87 studies were excluded from this review, a list of which can found in the references.

### 6.2 Risk of bias in included studies

A detailed summary of risk of bias for the 56 included studies is shown in Table 4. All 56 studies were utilised a cross sectional design and were rated using the JBI tool for cross‐sectional studies (The Joanna Briggs Institute, 2017, 2020). Studies were scored based on the number of items answered ‘yes’, with >70% yes = Low Risk of Bias, 50%–70% yes = Unclear Risk of Bias, and <50% ‘Yes’ = High Risk of Bias.

**Table 4:** Quality appraisal of included studies.

Study	Were the criteria for inclusion in the sample clearly defined and adhered to?	Was the sample included in the study representative of the population of interest?	Were the determinants measured in a valid and reliable way?	Were the behaviours measured in a valid and reliable way?	Were confounding factors/covariates identified?	Were strategies to deal with confounding factors/covariates stated and used?	Was appropriate statistical analysis used?	Is there evidence of selective reporting?	Overall Quality Rating
Al‐Sejari (2021)	Yes	No	Yes**—**scale	No**—**single item	Yes	Unclear	Yes	No	Low risk of bias
Al‐Shammary (2021)	Yes	Unclear	Yes**—**single item	Yes**—**single item	Yes	Yes	Yes	No	Low risk of bias
Al‐Wutayd (2021)	No	Yes	Yes**—**single item	Yes**—**single item	Unclear	Yes	Yes	No	Unclear risk of bias
Apanga (2021)	Yes	Unclear	Yes**—**single item	Yes**—**single item	Yes	Yes	Yes	No	Low risk of bias
Barrett (2021)	Yes	Unclear	Unclear	Yes**—**scale	Yes	Yes	Yes	No	Low risk of bias
Bogg (2020)	Yes	Unclear	Yes**—**scale	Yes**—**single item	Yes	Yes	Yes	No	Low risk of bias
Bruine de Bruin et al. (2020)	Yes	Yes	Yes**—**scale	Yes**—**scale	Yes	Yes	Yes	No	Low risk of bias
Bruine de Bruin et al. (2020)	No	Yes	Yes**—**scale	Unclear	Unclear	Unclear	Yes	No	High risk of bias
Callaghan (2021)	Yes	Yes	Yes**—**scale	Yes**—**scale	Yes	Yes	Yes	No	Low risk of bias
Cowling et al. (2020)	Yes	No	Yes**—**scale	Yes**—**scale	Yes	Yes	Yes	No	Low risk of bias
Dixon et al. (2022)	Yes	Yes	Yes**—**scale	Yes**—**scale	Yes	Yes	Yes	Yes	Low risk of bias
Dwipayanti (2021)	Unclear	No	Yes**—**scale	Yes**—**scale	Yes	Yes	Yes	No	Unclear risk of bias
Fujii (2021)	Unclear	Yes	Unclear	Unclear	Yes	Yes	Yes	No	Unclear risk of bias
Graupensperger (2021)	No	Unclear	Yes**—**scale	Yes**—**single item	No	No	Yes	No	High risk of bias
Haliwa et al. (2020)	No	Yes	Yes**—**scale	Yes**—**scale	Yes	Yes	Yes	No	Low risk of bias
Hsing (2021)	No	Yes	No**—**scale	Yes**—**scale	Yes	Yes	Yes	No	Unclear risk of bias
Iqbal (2021)	No	Unclear	Yes**—**single item	Yes**—**single item	Yes	No	Unclear	No	High risk of bias
Jang et al. (2020)	Yes	Yes	Yes**—**scale	Unclear	Yes	Yes	Yes	No	Low risk of bias
Jimenez (2020)	Yes	No	Yes**—**scale	Yes**—**scale	Yes	No	Unclear	No	Low risk of bias
Jovančević & Milićević (2020)	No	No	Yes**—**scale	Yes**—**scale	Yes	Yes	Yes	No	Unclear risk of bias
Kebede (2020)	No	No	Unclear	Unclear	Unclear	Unclear	Yes	No	High risk of bias
Kowalski (2020a) Study 1	Yes	Unclear	Yes**—**scale	Yes**—**single item	Yes	Yes	Yes	Yes	Low risk of bias
Kowalski (2020b) Study 2	Yes	Unclear	Yes**—**scale	Yes**—**single item	Yes	Yes	Yes	Yes	Low risk of bias
Lahiri (2021)	Yes	No	No**—**scale	Unclear	Yes	Unclear	Yes	Yes	High risk of bias
Lao et al. (2023)	Yes	Unclear	Yes**—**scale	Yes**—**single item	Yes	Yes	Yes	No	Low risk of bias
Lee (2020)	Yes	No	Yes**—**single item	Unclear	Unclear	Unclear	Yes	No	High risk of bias
Lee (2021)	Yes	Yes	Yes**—**scale	Yes**—**scale	Yes	Yes	Yes	Yes	Unclear risk of bias
Li (2021)	Unclear	Unclear	Yes**—**scale	Yes**—**scale	Yes	Yes	Yes	No	Unclear risk of bias
Matkovic et al. (2021)	No	Unclear	Yes**—**scale	Yes**—**scale	No	Unclear	Yes	No	Unclear risk of bias
Milman (2020)	Yes	No	Yes**—**scale	Unclear	Yes	Yes	Yes	Yes	Unclear risk of bias
Mousavi et al. (2022)	Yes	No	Yes**—**single item	Unclear	Yes	Yes	Yes	Yes	Unclear risk of bias
Nelson (2021)	Yes	No	Unclear	Yes**—**scale	Yes	No	Yes	No	Unclear risk of bias
Norman (2020)	Yes	Yes	Unclear	Yes**—**single item	Yes	Yes	Yes	No	Low risk of bias
Owhonda (2021)	No	Yes	No**—**scale	Yes**—**scale	Yes	Yes	Yes	Unclear	Unclear risk of bias
Lee et al. (2021)	Yes	Yes	Yes**—**scale	Yes**—**single item	Yes	Yes	Yes	No	Low risk of bias
Pal et al. (2020)	Yes	Unclear	Unclear	Unclear	Yes	Yes	Yes	No	Unclear risk of bias
Pan (2020)	No	Yes	Yes**—**single item	Yes**—**scale	Yes	Yes	Yes	No	Low risk of bias
Prete (2020)	No	No	Yes**—**scale	Yes**—**single item	Yes	No	Yes	No	Unclear risk of bias
Qian (2020)	Yes	Yes	Unclear	Yes**—**single item	Yes	Yes	Yes	No	Low risk of bias
Rattay (2021)	Unclear	Yes	Yes**—**single item	Unclear	Yes	Yes	Yes	No	Unclear risk of bias
Rui (2021) Study 1	Yes	Unclear	Yes**—**scale	Yes**—**scale	Yes	Yes	Yes	No	Low risk of bias
Rui (2021) Study 2	Yes	Unclear	Yes**—**scale	Yes**—**scale	Yes	Yes	Yes	No	Low risk of bias
Rui (2021) Study 3	Yes	Unclear	Yes**—**scale	Yes**—**scale	Yes	Yes	Yes	No	Low risk of bias
Rui (2021) Study 4	Yes	Unclear	Yes**—**scale	Yes**—**scale	Yes	Yes	Yes	No	Low risk of bias
Rui (2021) Study 5	Yes	Unclear	Yes**—**scale	Yes**—**scale	Yes	Yes	Yes	No	Low risk of bias
Rui (2021) Study 6	Yes	No	Yes**—**scale	Yes**—**scale	Yes	Yes	Yes	No	Low risk of bias
Sengeh (2020)	Yes	Unclear	No**—**scale	No**—**scale	Yes	No	Yes	Yes	High risk of bias
Sharma	Yes	No	Unclear	Yes**—**Scale	Yes	Yes	Yes	No	Unclear risk of bias
Shook (2020)	No	No	Yes**—**scale	Yes**—**scale	Yes	Yes	Yes	No	Unclear risk of bias
Si et al. (2021)	Yes	Yes	Yes**—**scale	Yes**—**single item	Yes	Yes	Yes	No	Low risk of bias
Souliotis (2021)	Yes	Yes	Unclear	Unclear	Yes	Yes	Yes	No	Unclear risk of bias
Stojanovic (2021)	Yes	No	Yes**—**scale	Yes**—**single item	Yes	Yes	Yes	No	Low risk of bias
Trifiletti (2021)	No	No	Yes**—**scale	Yes**—**single item	Yes	Yes	Yes	No	Unclear risk of bias
van den Broek‐Altenburg (2021)	Yes	Yes	Yes**—**scale	Yes**—**scale	Yes	Yes	Yes	No	Low risk of bias
Wang (2021)	Yes	Yes	Yes**—**scale	Yes**—**scale	Yes	No	Yes	No	Low risk of bias
Zewude et al. (2021)	Yes	Unclear	Yes**—**single item	Yes**—**single item	Yes	Yes	Yes	No	Low risk of bias

Overall, 30 studies were rated low risk of bias, 18 unclear risk of bias, and 8 were rated as high risk bias. Those studies deemed high risk of risk predominately received this rating due to lack of detail on measurement of handwashing and determinants (Bruine 2020; Kebede 2020) or the measure used was deemed not to be a reliable or valid measure (Sengeh 2020 (Sengeh et al., 2020). There was also poor reporting of study design and methodology (Graupensperger 2021; Iqbal 2021 (Iqbal & Younas, 2021); Kebede 2020; Lahiri 2021 (Lahiri et al., 2021); Lee 2020) and lack of sample demographics, making it difficult to determine representativeness (Graupensperger 2021; Iqbal 2021). In three studies it was evident that the sample was not representative (Kebede 2020; Lahiri 2021; Lee 2020).

### 6.3 Data and analysis

#### 6.3.1 Meta‐analysis

In total we analysed 52 effect sizes across 6 determinant groups, and included 28 studies. The summary effect of each determinant group can be seen in the Summary of findings table 1 along with 95% confidence intervals (CIs) and heterogeneity statistics. As shown in the summary of findings table, our analyses indicate significant relationships between knowledge about behaviour and disease, social norms, COVID‐related worry and anxiety and handwashing behaviour. There is no significant relationship observed between perceived severity and handwashing or between perceived susceptibility and handwashing. All data is reported in Tables 5, 6, 7.

**Table 5:** Handwashing and anxiety and worry.

Study	*n*	Description of determinant			Effect size	CI
*Anxiety*						
Milman (2020)	408	COVID anxiety	Unadjusted	*r*	0.32	
Kowalski (2020a) S1	507	Coronavirus‐related anxiety	Unadjusted	*r*	0.42	
Kowalski (2020b) S2	840	Coronavirus‐related anxiety	Unadjusted	*r*	0.18	
*Worry*						
Prete (2020)	618	Worry	Unadjusted	*d*	1.02	
Jang et al. (2020)	1004	Worried about disease	Unadjusted	OR	4.25	(3.10 −5.85)
Jimenez (2020)	290	Worry	Unadjusted	OR	0.16	(0.09, 0.23)
Al‐Sejari (2021)	1413	Worry about illness	Unadjusted	*r*	0.239	

Abbreviation: CI, confidence interval.

**Table 6:** Handwashing and cognition.

Study	*n*	Description of determinants			Effect size	CI
*Perceived control*						
Lao et al. (2023)	229	Action control	Unadjusted	*r*	0.28	
Bogg (2020)	500	Perceived control	Unadjusted	*r*	0.22	
Trifiletti (2021)	248	Perceived behavioural control of HW	Unadjusted	*r*	0.06	
Norman (2020)	477	Autonomy	Unadjusted	*r*	0.17	
*Attitudes towards behaviour*						
Trifiletti (2021)	248	Attitude towards handwashing	Unadjusted	*r*	0.22	
Barrett (2021)	292	Attitudes	Unadjusted	*r*	0.26	(0.15, 0.37)
Bogg (2020)	500	Attitudes	Unadjusted	*r*	0.29	
Norman (2020)	477	Attitude towards handwashing	Unadjusted	*r*	0.37	
Norman (2020)	477	Injunctive norms	Unadjusted	*r*	0.30	
Matkovic et al. (2021)	344	Attitudes towards handwashing	Unadjusted	*r*	0.41	
Al‐Wutayd (2021)	1323	Attitudes towards handwashing behaviour	Unadjusted	*t*	0.417	
*Perceived self‐efficacy*						
Lee et al. (2021)		Perceived self‐efficacy	Unadjusted	OR	1.19	(0.88, 1.61)
Kebede (2020)	247	Efficacy—self‐control	Unadjusted	OR	3.54	(1.9, 6.57)
Barrett (2021)	293	Self‐efficacy—infection avoidance	Unadjusted	*r*	−0.08	(−0.2, 0.04)
Barrett (2021)	293	Self‐efficacy—hand hygiene	Unadjusted	*r*	0.38	(0.27, 0.48)
Bogg (2020)	500	Self‐efficacy	Unadjusted	*r*	0.28	
Norman (2020)	477	Capacity	Unadjusted	*r*	0.33	
Lao et al. (2023)	229	Action self‐efficacy	Unadjusted	*r*	0.22	
*Perceived effectiveness of behaviour*						
Lee et al. (2021)	897	Perceived efficacy of anti‐COVID strategies	Unadjusted	OR	2.66	(2.02, 3.51)
Al‐Shammary (2021)	400	Perceived effectiveness of preventive measures	Unadjusted	SMD	0.355	
Sharma et al. (2021)	713	Advantages of behaviour	Unadjusted	SMD	0.23	
*Perceived risk*						
Barrett (2021)	293	Risk perception	Unadjusted	*r*	0.2	(0.1, −0.29)
Bogg (2020)	500	Perceived risk of exposure	Unadjusted	*r*	0.05	
Bogg (2020)	500	Perceived risk of health consequence	Unadjusted	*r*	0.17	
Trifiletti (2021)	248	risk perception	Unadjusted	*r*	0.26	
Lao et al. (2023)	229	Risk perception	Unadjusted	*r*	0.16	
Al‐Shammary (2021)	400	Risk perception	Unadjusted	Mean (SD)	6.9 (3.2), 5.61 (2.21)	
*Perceived severity*						
Bruine & Bennett (2020)	6684	Risk of dying if infected	Unadjusted	*r*	0.01	
Bruine de Bruin et al. (2020)	5517	Risk of Dying if infected	Unadjusted	*r*	0	
Lee et al. (2021)	897	Likelihood of requiring ICU admission with COVID	Unadjusted	OR	1.05	(0.81, 1.37)
Lee et al. (2021)	897	Perceived disease severity	Unadjusted	OR	0.75	(0.58, 0.98)
*Perceived susceptibility*						
Haliwa et al. (2020)	353	Perceived likelihood of contracting COVID‐19	Unadjusted	*r*	−0.02	
Bruine (2020)	5517	Risk of getting infected	Unadjusted	*r*	0.03	
Si et al. (2021)	1019	Perceived infectability	Unadjusted	*r*	−0.1	
Bruine de Bruin et al. (2020)	6684	Perceived susceptibility	Unadjusted	*r*	0.11	
Norman (2020)	477	Perceived susceptibility	Unadjusted	*r*	0.18	
*Social norms*						
Bogg (2020)	500	Perceived norms	Unadjusted	*r*	0.29	
Graupensperger (2021)	539	Perceived peer norms	Unadjusted	*r*	0.49	
Graupensperger (2021)	539	Perceived peer norms	Unadjusted	*r*	0.47	
Trifiletti (2021)	248	Social norms about handwashing	Unadjusted	*r*	0.13	
Norman (2020)	477	Injunctive norms	Unadjusted	*r*	0.23	
Norman (2020)	477	Descriptive norms	Unadjusted	*r*	0.13	

Abbreviation: CI, confidence interval.

**Table 7:** Handwashing and knowledge.

Study	*n*	Description of determinants			Effect size	CI
*Knowledge of behaviour*						
Barrett (2021)	290	Hand hygiene effectiveness knowledge	Unadjusted	*r*	−0.07	(0.77, 0.95)
Al‐Wutayd (2021)	1323	Knowledge of handwashing	Unadjusted	*r*	0.375	(0.33, 0.42)
Apanga (2021)	527	Knowledge of behaviours to prevent COVID infection	Unadjusted	*r*	0.435	(0.36, 0.50)
*Knowledge of disease*						
Barrett (2021)	287	Knowledge	Unadjusted	*r*	0.1	
Iqbal (2021)	1789	COVID knowledge	Unadjusted	*r*	0.418	(0.37, 0.46)
Pal et al. (2020)	212	Knowledge of disease	Unadjusted	OR	1.07	(0.97, 1.18)
Sengeh (2020)	1253	Knowledge about COVID (low vs high)	Unadjusted	OR	6.33	(2.98, 13.45)
Apanga (2021)	527	Knowledge about COVID symptoms and transmission	Unadjusted	*r*	0.279	
Owhonda (2022)	1294	Knowledge about COVID (poor vs good)	Unadjusted	OR	1.16	(1.1, 1.24)

Below we present forest plots (Figures 2, 3, 4, 5, 6, 7, 8, 9, 10, 11, 12, 13) for each determinant and interpret these findings further.

**Figure 2:**
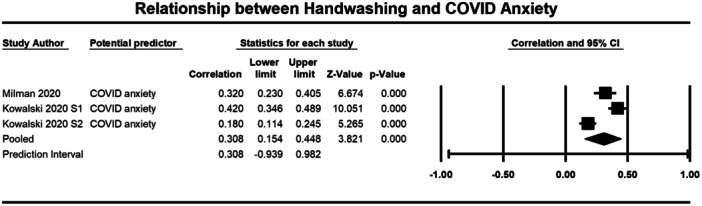
Relationship between handwashing and COVID anxiety. CI, confidence interval.

**Figure 3:**
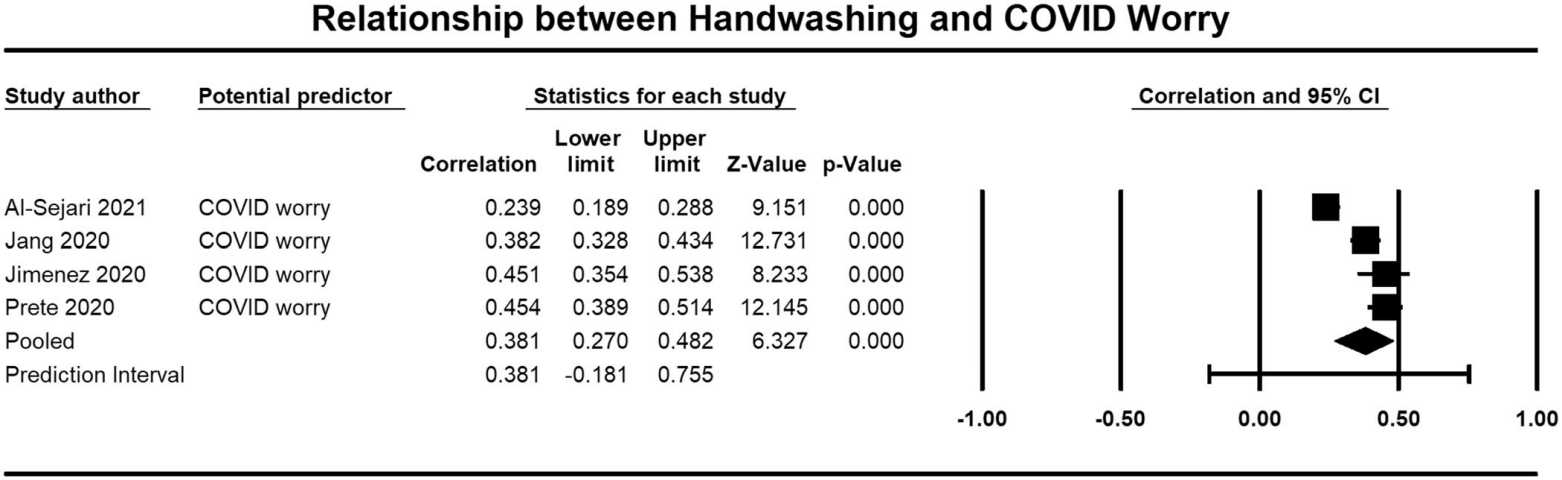
Relationship between handwashing and COVID worry. CI, confidence interval.

**Figure 4:**
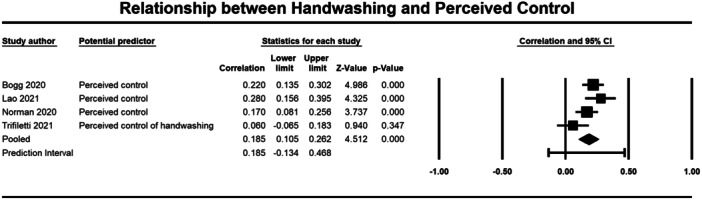
Relationship between handwashing and percieved control. CI, confidence interval.

**Figure 5:**
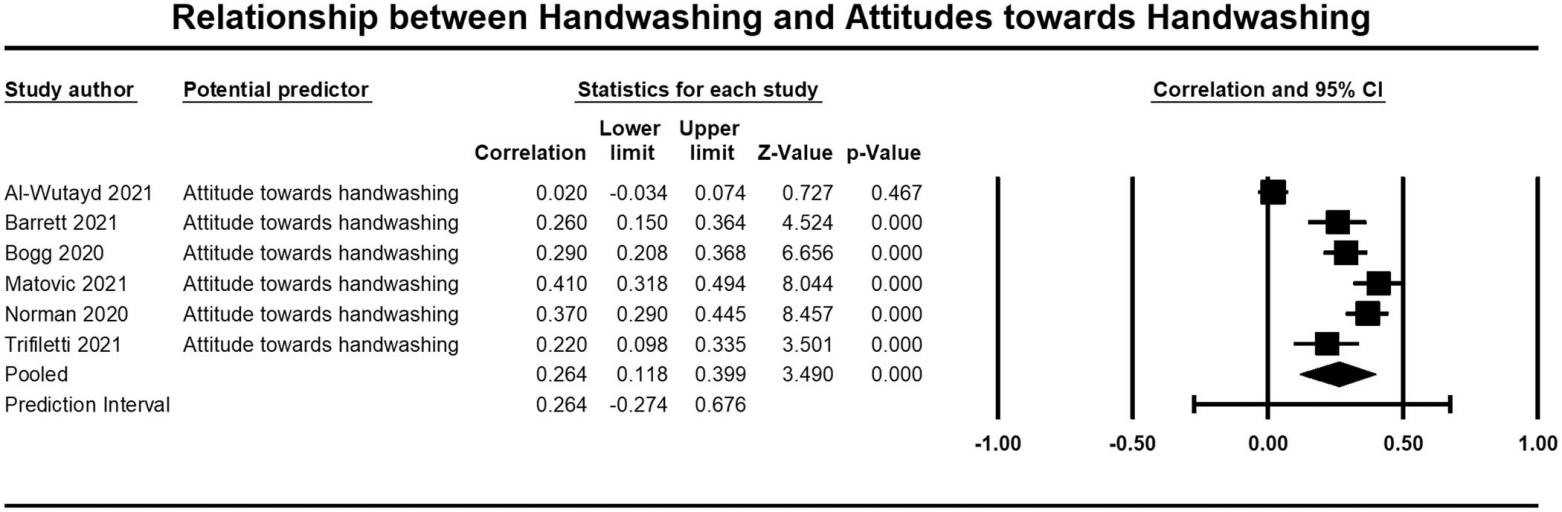
Relationship between handwashing and attitude towards handwashing. CI, confidence interval.

**Figure 6:**
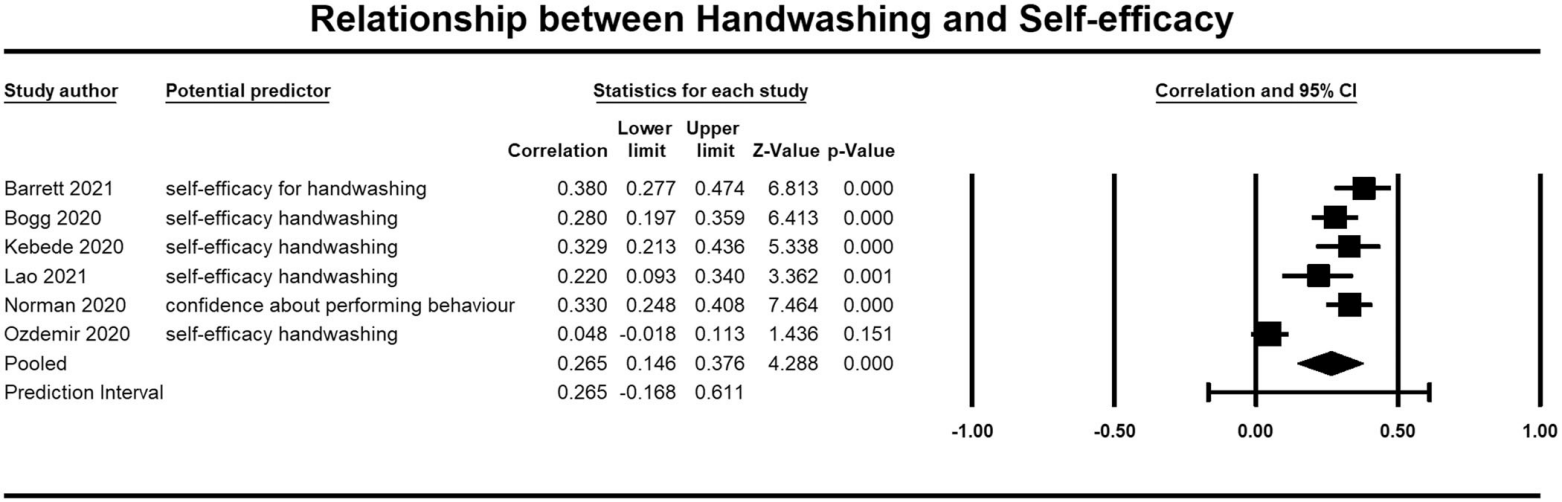
Relationship between handwashing and self‐efficacy. CI, confidence interval.

**Figure 7:**
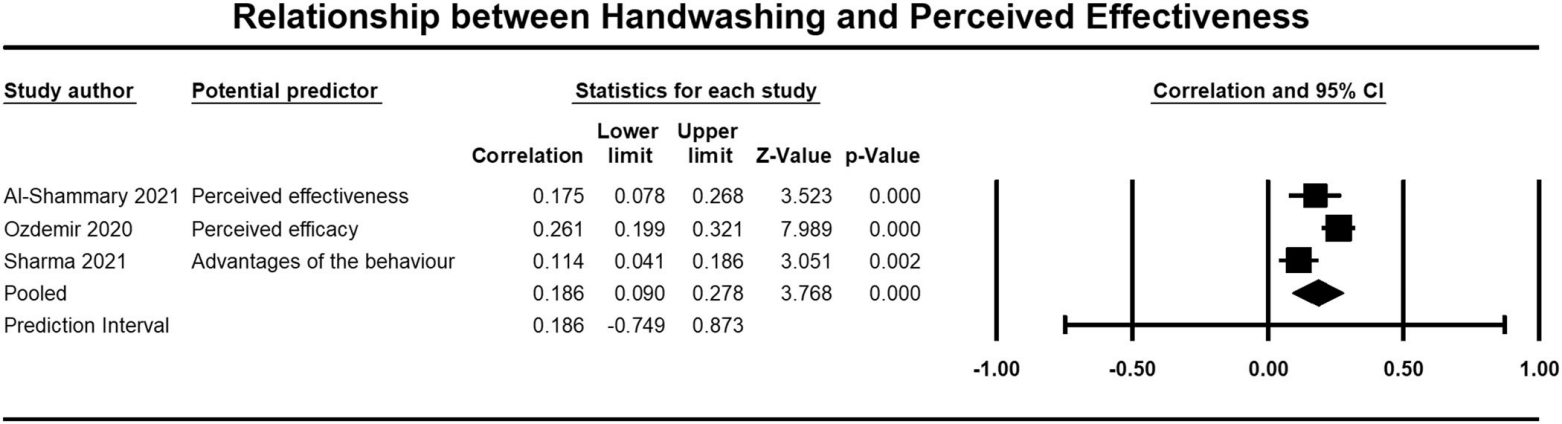
Relationship between handwashing and perceieved effectiveness. CI, confidence interval.

**Figure 8:**
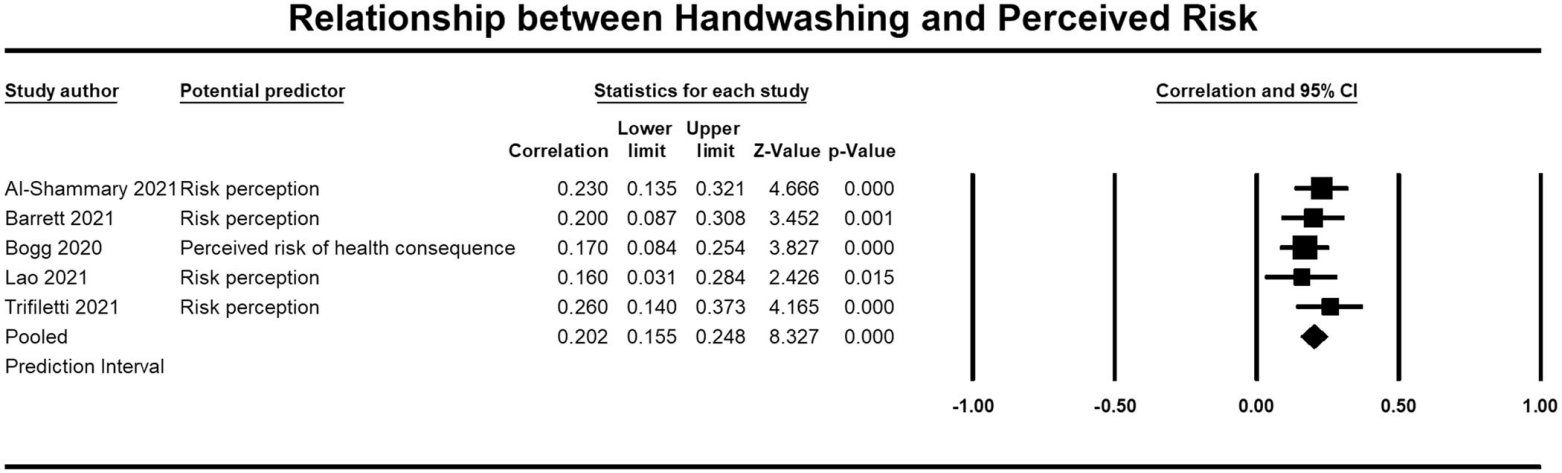
Relationship between handwashing and perceieved risk. CI, confidence interval.

**Figure 9:**
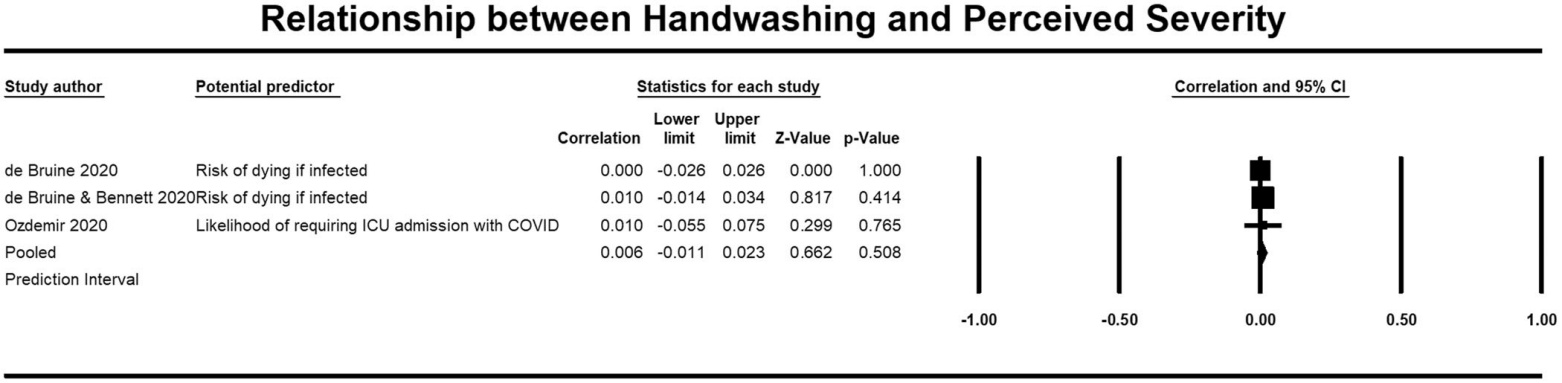
Relationship between handwashing and perceived severity. CI, confidence interval.

**Figure 10:**
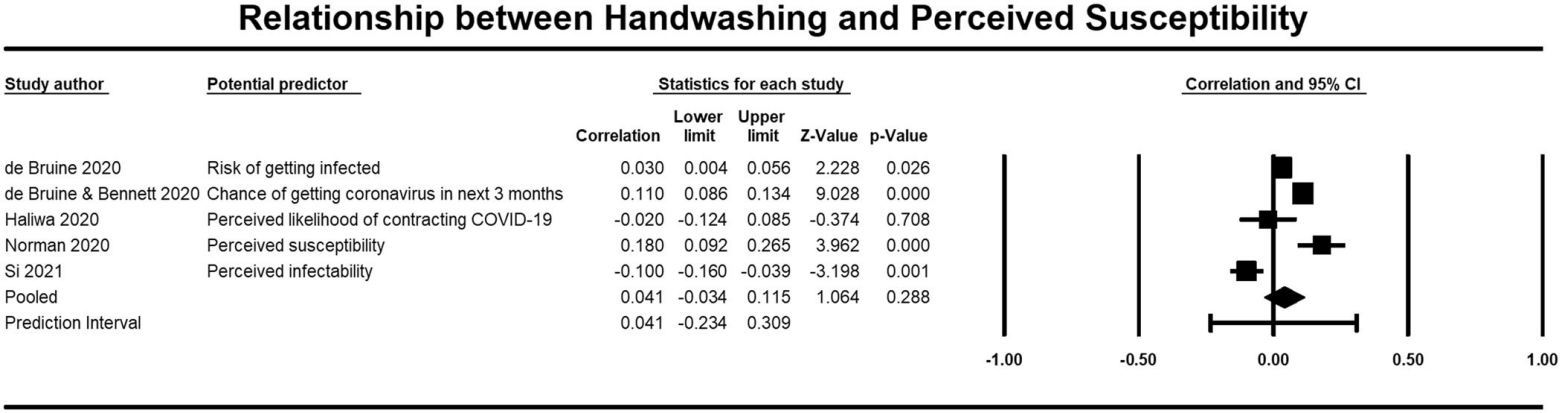
Relationship between handwashing and perceived susceptibility. CI, confidence interval.

**Figure 11:**
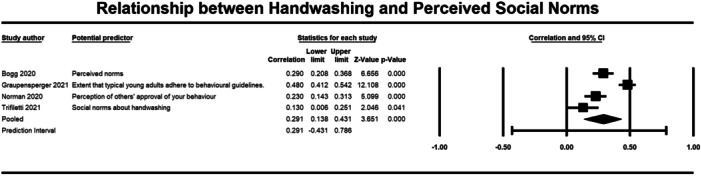
Relationship between handwashing and social norms. CI, confidence interval.

**Figure 12:**
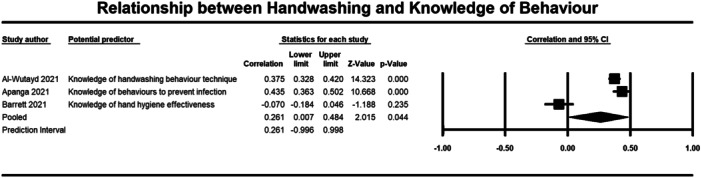
Relationship between handwashing and knowledge of behaviour. CI, confidence interval.

**Figure 13:**
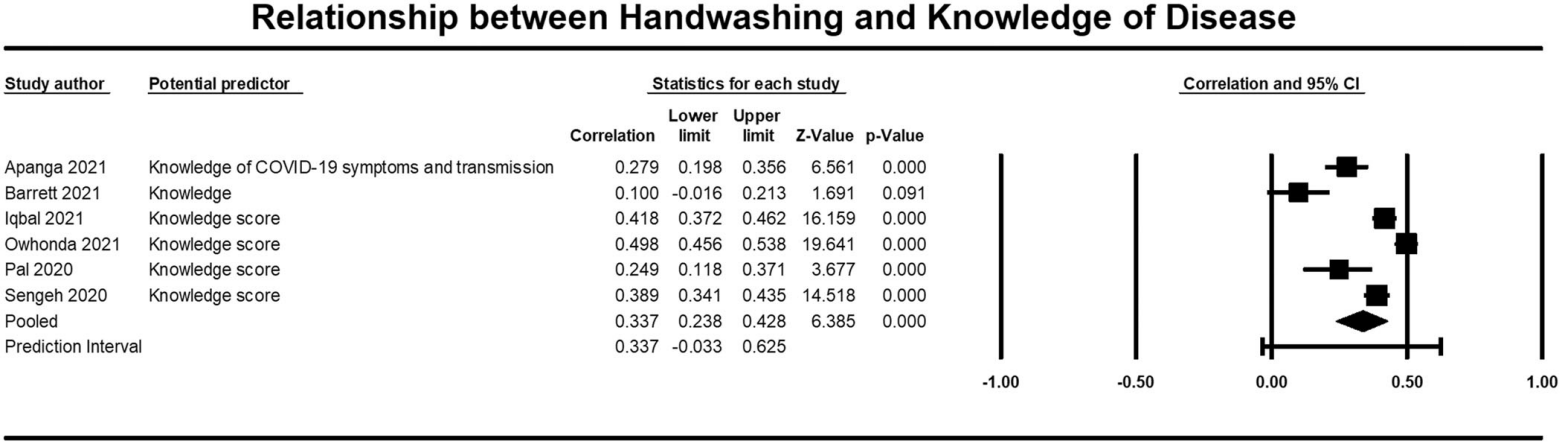
Relationship between handwashing and knowledge of disease. CI, confidence interval.

##### Emotions

Three studies were included in the meta‐analysis that examined the relationship between COVID‐related anxiety and handwashing behaviour (1755 participants) (Kowalski 2020a; Kowalski 2020b; Milman 2020 (Milman, 2020). The average correlation between COVID related anxiety and handwashing across the three studies was moderate (*r* = 0.308, 95% CI = 0.154, 0.448), but was significant (*p* ≤ 0.001) (Figure 2). There was significant heterogeneity across studies (*τ*² = 0.019; *Q* = 22.981, *df* = 2; *p* ≤ 0.001; *I*² = 91%). There were similar results for COVID‐related worry (3325 participants). With a moderate and significant correlation observed across the four included studies (*r* = 0.381, 95% CI = 0.270, 0.482, *p* ≤ 0.001) (Al‐Sejari 2021; Jang et al. 2020 (Jang et al., 2020); Jimenez 2020 (Jimenez et al., 2020); Prete 2020 (Prete et al., 2020) (Figure 3). Again, there was significant heterogeneity across studies (*τ*² = 0.014; *Q* = 35.762, *df* = 3; *p* ≤ 0.001; *I*² = 92%). These results indicate that experiencing more COVID‐related anxiety and worry was significantly correlated to handwashing behaviour.

##### Cognitions

The meta‐analysis included four studies which examined the relationship between perceived control (1454 participants) and handwashing (Bogg 2020; Lao et al. 2023; Norman 2020; Trifiletti 2021). Results showed a weak but significant relationship (*r* = 0.185, 95% CI = 0.105, 0.262, *p* ≤ 0.001), with no significant heterogeneity across studies (*τ*² = 0.004; *Q* = 7.013, *df* = 3, *p* = 0.071; *I*² = 57%) (Figure 4).

A weak but significant (*p* ≤ 0.001) relationship was also observed between attitudes (3184 participants) (*r* = 0.264, 95% CI, 0.118, 0.399), self‐efficacy (2643 participants) (*r* = 0.265, 95% CI = 0.146, 0.376), perceived effectiveness (2010 participants) (*r* = 0.186, 95% CI = 0.090, 0.278), perceived risk (1670 participants) (*r* = 0.202, 95% CI = 0.155, 0.248), and social norms (1764 participants) (*r* = 0.291, 95% CI = 0.138, 0.431) and handwashing behaviour (Figures 5, 6, 7, 8, 9). Heterogeneity was significant across all these determinants. For the meta‐analysis of attitudes, Norman (2020) provided two effect sizes: one for experiential attitudes (*r* = 0.44) and one for instrumental attitudes (*r* = 0.30). We used an average of these two estimates in the meta‐analysis. For the meta‐analysis of perceived effectiveness, Al‐Shammary (2021) provided four effect sizes for the relationship between handwashing and perceived effectiveness of preventive measures in the marketplace (*r* = 0.26), in the workplace (*r* = 0.17), in healthcare settings (*r* = 0.10), and in travel settings (*r* = 0.16). We used the average of these estimates in the meta‐analysis. In the case of social norms, Graupensperger (2021) provided correlations between social norms and handwashing with soap and water (*r* = 0.49) and also using hand sanitiser (*r* = 0.47). We used the average of these two correlations in the meta‐analysis.

Both perceived severity (13,098 participants) and susceptibility (14,050 participants) had a non‐significant correlation with handwashing behaviour (*r* = 0.006, 95% CI = −0.011, 0.023; *r* = 0.041, 95% CI = −0.034 to 0.115, respectively) (Figures 10 and 11).

##### Knowledge

Seven studies were included in the meta‐analysis that examined the relationship between knowledge of behaviour (2140 participants) and disease (4893 participants) and handwashing behaviour (Al‐Wutayd 2021; Apanga 2021; Barrett 2021; Iqbal 2021; Owhonda 2022; Pal et al., 2020; Sengeh 2020). For knowledge of behaviour the average correlation with handwashing was small (*r* = 0.261, 95% CI = 0.007, 0.484, *p* = 0.044) (Figure 12). To conduct this meta‐analysis, we averaged the correlations provided by Al‐Wutyad (2021) and we averaged the correlations provided by Apanga (2021). Al‐Wutyad (2021) had provided correlations between handwashing and knowledge about recommendations around handwashing that comprised knowledge about: following the correct technique (*r* = 0.08), duration of handwashing (*r* = 0.21), handwashing after visiting public places (*r* = 0.88), and handwashing after touching high touch surfaces outside (*r* = 0.33). Apanga (2021) provided correlations between handwashing and knowledge about behaviours that reduce COVID‐19 infection, including avoiding touching the T‐zone (*r* = 0.46) and avoiding crowded places (*r* = 0.41).

For knowledge of disease, a moderate average correlation was found with handwashing across the studies (*r* = 0.337, 95% CI = 0.238, 0.428, *p* ≤ 0.001) (Figure 13). Apanga (2021) provided correlations between handwashing and knowledge of: COVID symptoms (*r* = 0.29), transmission via respiratory droplets (*r* = 0.09), and transmission via touching contaminated surfaces (*r* = 0.45). We used the average correlation from this study in the meta‐analysis.

There was significant heterogeneity for both knowledge of behaviour and disease (*τ*² = 0.051; *Q* = 60.292, *df* = 2; *p* ≤ 0.001; *I*² = 97%; *τ*² = 0.016, *Q* = 65.716, *df* = 5; *p* = <0.001; *I*² = 92%, respectively). These results indicate that having more knowledge about behaviours and COVID was significantly correlated to handwashing behaviour.

#### 6.3.2 Narrative synthesis of results

A total of 34 studies were included in the narrative synthesis. Details of the individual studies that contribute to this synthesis are show in Table 3.

##### Barriers influencing handwashing behaviour

Four studies (Barrett 2021; Dwipayanti 2021 (Dwipayanti Ni Made et al., 2021); Hsing 2021; Li 2021) examined the relationship between perceived barriers and handwashing behaviour Table 8. One study (Hsing 2021) recruited samples from four different counties and found generally weak associations between barriers and using either hand sanitiser or hand soap. In participants living in one of these locations (Hong Kong), a larger effect was observed. However, it should be noted that the sample size in this location was much smaller (around 1200 participants) in comparison to the other countries where participants were recruited from the USA, Mexico and Taiwan, which had sample sizes ranging from approximately 3000 to 640,000). Weak associations were also found in the studies of Dwipayanti 2021 and Li 2021.

**Table 8:** Handwashing and perceived barriers.

Study ID	Determinant	Effect size (CI)	*n*
Hsing (2021)	Perceived barriers to using hand sanitiser (USA)	AOR: 0.88 (0.74–1.03)	3070
	Perceived barriers to using hand sanitiser (Mexico)	AOR: 1.01 (0.88–1.15)	3946
	Perceived barriers to using hand sanitiser (Hong Kong)	AOR: 1.14 (0.74–1.77)	1201
	Perceived barriers to using hand sanitiser (Taiwan)	AOR: 0.86 (0.79–0.94)	63,634
	Perceived barriers to using hand soap (USA)	AOR: 0.73 (0.49–1.07)	3070
	Perceived barriers to using hand soap (Mexico)	AOR: 1.35 (0.98–1.87)	3946
	Perceived barriers to using hand soap (Hong Kong)	AOR: 7.59 (1.88–53.9)	1201
	Perceived barriers to using hand soap (Taiwan)	AOR: 1.01 (0.81–1.27)	63,634
Dwipayanti (2021)	Perceived barriers	AOR: 1.3 (0.9–1.9)	
Li et al. (2021)	Perceived barriers	Adjusted regression coefficient: 0.00 (−0.03 to 0.02)	326
Barrett (2021)	Perceived barrier (focused on time)	*r* = 0.49 (0.4–0.58)	293
		OR^a^ = 7.68 (4.87–13.23)	

Abbreviations: AOR, adjusted odds ratio; CI, confidence interval; OR, odds ratio.

a Converted to OR to allow comparison with other studies in the table.

##### COVID‐19‐related fear or worry

Weak, but positive associations were reported in three studies (Al‐Shammary 2021; Jovančević & Milićević 2020; Rattay 2021 (Petra et al., 2021) which examined the role of fear on handwashing, supporting the contention that these behaviours may be driven or motivated by the level of COVID‐19 related fear (Table 9). Worry or concern about COVID‐19 was also consistently found to be associated with handwashing behaviours in five of the included studies (Al‐Sejari 2021; Callaghan 2021; Rattay 2021; Shook 2020 (Shook Natalie et al., 2020); Nelson 2021 (Nelson Tracy et al., 2021), but again, the reported effect sizes for these associations were weak (Table 10). One of these studies (Nelson 2021) did not report any differences based on whether handwashing behaviour were being carried out in or outside of work contexts.

**Table 9:** Handwashing and fear.

Study ID	Determinant	Effect size (CI)	*n*
Rattay (2021)	Fear	AOR: 1.05 for women, 1.03 for men	13,430 women, 13,037 men
Jovancevic & Milićević (2020)	Fear of others being infected Fear of self‐being infected	Multiple regression coefficients: 0.02 in Latin America 0.13 in Serbia 0.23 in Latin America 0.14 in Serbia	412 Latin America; 120 Sebia
Al‐Shammary (2021)	Fear of COVID‐19	Level of fear was higher in those who engaged in hand hygiene procedures: *M* (SD) yes = 7.42 (2.91), no = 6.59 (3.08) Cohen's *d* = 0.28 OR* = 1.66	400

Abbreviations: AOR, adjusted odds ratio; CI, confidence interval; OR, odds ratio﻿﻿.

* *p* < 0.05.

**Table 10:** Handwashing and worry and concern.

Study ID	Determinant	Effect size (CI)	*n*
Rattay (2021)	Worries	AOR: 1.14 for women, 1.14 for men	13,430 women; 13,037 men
Callaghan (2021)	COVID worry	AOR: 1.28 (1.07–1.52)	5009
Al‐Sejari (2021)	Concern about pain	*r* = 0.148 OR* = 1.72	1413
Shook (2020)	COVID‐19 concern	Multiple regression coefficient: 0.13	1019
Nelson (2021)	Level of concern about contracting COVID‐19 Level of concern about exposing others to COVID‐19	*r* = 0.14 (washing hands at work), OR* = 1.67 *r* = 0.17 (washing hands outside of work), OR* = 1.87 *r* = 0.10 (washing hands at work), OR* = 1.44 *r* = 0.13 (washing hands outside of work), OR* = 1.61	508

Abbreviations: AOR, adjusted odds ratio; CI, confidence interval; OR, odds ratio﻿﻿﻿﻿.

* *p* < 0.05.

##### Perceived susceptibility to COVID‐19 and severity of COVID‐19

Findings related to the influence of perceived susceptibility on handwashing or hand sanitising behaviours were mixed across the 14 included studies (Cowling et al., 2020 (Cowling et al., 2020); Dwipayanti 2021; Fujii 2021; Hsing 2021; Lahiri 2021; Lee 2020; Lee et al. (2021); Mousavi et al. 2022 (Mousavi et al. 2022); Pan 2020; Rui et al., 2021; (Rui et al., 2021); Qian 2020 (Mengcen et al., 2020); Rattay 2021; Shook 2020; Zewude et al. 2021). While the majority of these reported weak effect sizes, the direction of these effects varied, with both positive and negative associations being observed (Table 11). Two studies (Dwipayanti 2021; Mousavi et al., 2022) which recruited participants in Indonesia and Afghanistan, respectively, did report larger associations between variables. One of these (Mousavi et al., 2022), differed from other studies in that it examined the likelihood of family members getting infected, and included a relatively small sample size. In comparison to other evidence included in the analysis, both of these studies had larger confidence intervals around the reported odds ratios.

**Table 11:** Handwashing and perceived susceptibility.

Study ID	Determinant	Effect size (CI)	*n*
Shook (2020)	Perceived infectability	Multiple regression coefficient: −0.04	1019
Lee (2020)	Perceived susceptibility	Multiple regression coefficient: 0.03 (−0.01 to 0.08)	973
Lee et al. (2021)	Perceived susceptibility	Multiple regression coefficient: 0.02	990
Hsing (2021)	Perceived susceptibility of infection: USA Mexico Hong Kong Taiwan	AOR: 1.12 (0.95–1.33) AOR: 1.23 (1.06–1.42) AOR: 1.44 (1.11–1.87) AOR: 1.08 (1.04–1.12)	3070 3946 1201 63,634
Rattay (2021)	Perceived susceptibility	AOR: 0.97 (women), 0.99 (men)	13,430 women; 13,037 men
Dwipayanti (2021)	Perceived susceptibility	AOR: 6.34 (2.28–17.62)	896
Pan (2020)	Perceived risk of contracting COVID‐19	AOR: 0.58 (0.50–0.68)	3035
Cowling et al. (2020)	Those with thought they were likely to contract the virus Those with thought they were unlikely to contract the virus Those with thought they were very likely to contract the virus Those with thought they were very unlikely to contract the virus Those with thought they would never contract the virus	AORs: Handwashing after returning home: 1.12 (0.91–1.38) Handwashing after sneezing: 0.93 (0.80–1.09) Handwashing after touching common objects: 1.01 (0.87–1.18) Use of liquid soap: 0.96 (0.82–1.14) Handwashing after returning home: 0.82 (0.71–0.95) Handwashing after sneezing: 0.91 (0.81–11.03) Handwashing after touching common objects: 0.95 (0.85–1.06) Use of liquid soap: 0.93 (0.82–1.05) Handwashing after returning home: 0.86 (0.45–1.63) Handwashing after sneezing: 1.15 (0.68–1.94) Handwashing after touching common objects: 1.67 (1.04–2.67) Use of liquid soap: 1.47 (0.79–2.74) Handwashing after returning home: 0.87 (0.69–1.10) Handwashing after sneezing: 0.99 (0.81–1.20) Handwashing after touching common objects: 0.94 (0.79–1.13) Use of liquid soap: 0.98 (0.81–1.18) Handwashing after returning home: 0.88 (0.71–1.09) Handwashing after sneezing: 1.06 (0.89–1.27) Handwashing after touching common objects: 0.99 (0.84–1.16) Use of liquid soap: 1.00 (0.83–1.19)	12,965
Mousavi et al. (2022)	High likelihood of family getting infected	OR = 3.63 (1.89–6.98)	64
Lahiri (2021)	Higher vulnerability of the participants to COVID‐19, with progression of pandemic (time) Higher vulnerability of a respondent to COVID‐19 in comparison to others Higher vulnerability of the participants to COVID‐19, due to current residence area Higher vulnerability of the participants to COVID‐19, with progression of pandemic (time) Higher vulnerability of a respondent to COVID‐19 in comparison to others Higher vulnerability of the participants to COVID‐19, due to current residence area	Adjusted prevalence ratios: Frequently washing hands with soap and water 1.01 (1.00–1.02) 1.03 (1.01–1.05) 1.00 (0.97–1.02) Frequently cleaning hands with sanitizer 0.92 (0.91–0.94) 1.21 (1.14–1.28) 1.13 (1.10–1.16)	2615 2617
Fujii (2021)	Perceived susceptibility China Italy Japan Korea UK USA	AORs: 0.81 (0.71–0.91) 0.99 (0.84–1.18) 0.98 (0.90–1.09) 0.77 (0.66–0.90) 1.05 (0.89–1.27) 0.91 (0.80–1.03)	994 1020 981 918 994 1038
Zewude et al. (2021)	Think can be infected by COVID‐19	AOR: 0.68 (0.35–1.32)	379
Qian (2020)	Likelihood of contracting virus	AOR: 0.8 (0.7–1.0)	1011
Rui (2021) Study 5	Perceived susceptibility	AOR 0.92 (0.73–1.16)	329
Rui (2021) Study 4	Perceived susceptibility	AOR 0.86 (0.69–1.07)	343
Rui (2021) Study 3	Perceived susceptibility	AOR 0.85 (0.69–1.05)	315
Rui (2021) Study 2	Perceived susceptibility	AOR 0.93 (0.73–1.19)	319
Rui (2021) Study 1	Perceived susceptibility	AOR 1.01 (0.79–1.3)	321
Rui (2021) Study 6	Perceived susceptibility	AOR 0.92 (0.67–1.25)	315

Abbreviations: AOR, adjusted odds ratio; CI, confidence interval; OR, odds ratio.

Similar findings were also observed for perceived severity of COVID‐19, with 18 studies (Dwipayanti 2021; Fujii 2021; Hsing 2021; Lahiri 2021; Lee 2020; Lee et al. 2021; Mousavi et al., 2022; Pan 2020; Qian 2020; Rattay 2021; Rui et al., 2021; Zewude et al. 2021) finding associations that were in both directions but which had weak effect sizes (Table 12).

**Table 12:** Handwashing and perceived severity.

Study ID	Determinant	Effect size (CI)	*n*
Lee (2020)	Perceived severity	Multiple regression coefficient: 0.08 (0.03–0.13)	973
Lee et al. (2021)	Perceived susceptibility	Multiple regression coefficient: 0.05	990
Hsing (2021)	Perceived severity of COVID‐19: USA Mexico Hong Kong Taiwan	AOR: 0.33 (1.09–1.61) AOR: 1.06 (0.91–1.22) AOR: 1.22 (0.90–1.65) AOR: 1.24 (1.20–1.29)	3070 3946 1201 63,634
Rattay (2021)	Perceived severity	AOR: 1.17 (women), 1.14 (men)	13,430 women; 13,037 men
Dwipayanti (2021)	Perceived severity**—**fatal	AOR: 1.06 (0.38–2.96)	896
Pan (2020)	Perceived severity of COVID‐19	AOR: 1.03 (0.99–1.07)	3035
Cowling et al. (2020)		AORs:	12,965
Mousavi et al. (2022)	Low likelihood of survival if infected	OR = 2.01 (1.09–3.69)	64
Lahiri (2021)	Higher perceived severity of the disease compared to existing reports Higher perceived severity of the disease compared to existing reports	Adjusted prevalence ratios: Frequently washing hands with soap and water 1.01 (0.98–1.04) Frequently cleaning hands with sanitizer 0.91 (0.86–0.97)	2615 2617
Fujii (2021)	Perceived severity China Italy Japan Korea UK USA	AORs: 1.02 (0.93–1.13) 1.05 (0.90–1.25) 0.96 (0.88–1.06) 1.12 (0.98–1.30) 1.01 (0.85–1.21) 0.92 (0.81–1.06)	994 1020 981 918 994 1038
Zewude et al. (2021)	Think you will die if infected by COVID‐19	AOR: 0.74 (0.39–1.41)	379
Qian (2020)	Perceived severity	AOR: 0.9 (0.8–1.1)	1011
Rui (2021) Study 1	Perceived severity	AOR 0.75 (0.96–1.93)	321
Rui (2021) Study 2	Perceived severity	AOR 1.36 (1.22–2.35)	319
Rui (2021) Study 3	Perceived severity	AOR 1.69 (0.67–1.31)	315
Rui (2021) Study 4	Perceived severity	AOR 0.94 (1.1–1.9)	343
Rui (2021) Study 5	Perceived severity	AOR 1.45 (1.07–2.1)	329
Rui (2021) Study 6	Perceived severity	AOR 1.5 (1.03–1.81)	315

Abbreviations: AOR, adjusted odds ratio; CI, confidence interval; OR, odds ratio.

##### Other Beliefs and motivations about COVID‐19

Ten studies (Al‐Shammary 2021; Apanga 2021; Dixon et al. 2022 (Dixon et al., 2022); Kowalski 2020b; Norman 2020; Souliotis 2021 (Kyriakos et al., 2021); Stojanovic 2021 (Jovana et al., 2021); van den Broek‐Altenburg; Wang 2021; Zewude et al. 2021) were included in this category, which included views on the need for control measures, on how similar COVID‐19 is to influenza, and on which factors influenced people to followed preventative recommendations (Table 13). Like the relationships between handwashing and susceptibility, or severity of COVID‐19, the effect sizes found here were weak. Studies in this section did suggest that handwashing was more likely when people held beliefs that COVID‐19 should be taken more seriously. The strongest associations were found for motivations that were around protecting others. For example, it was observed that while self‐protection was a predictor of handwashing, protecting family members and the general public, was more strongly associated with these behaviours (Stojanovic 2021; van den Broek‐Altenburg).

**Table 13:** Handwashing and beliefs and motivations about COVID‐19.

Study ID	Determinant	Effect size (CI)	*n*
Al‐Shammary (2021)	Misconceptions about COVID‐19	Level of misconceptions was higher in those who did not engage in hand hygiene procedures: *M*(SD) yes = 8.01 (2.43), no = 9.09 (3.98) Cohen's *d* = 0.33 OR* = 1.82	Yes: 366, No: 34
Souliotis (2021)	Perceiving the virus to be airborne neutral vs. agree disagree vs. agree Perception that the virus is similar to common flu neutral vs. agree disagree vs. agree Perception that the virus may be asymptomatic neutral vs. agree disagree vs. agree Perception that the virus is dangerous for older people and for those with underlying health problems neutral vs. agree disagree vs agree Perceived control ‘the virus is out of control’ neutral vs. agree disagree vs. agree	AORs: 0.83 (0.61–1.12) 0.61 (0.48–0.78) 1.33 (0.92–1.94) 1.65 (1.22–2.24) 0.75 (0.4–1.38) 0.71 (0.22–2.28) 0.83 (0.62–1.12) 1.12 (0.75–1.66) 0.95 (0.67–1.34) 0.63 (0.46–0.88)	923
Apanga (2021)	No need for preventive measures, COVID‐19 is not deadly	AOR: 0.6 (0.4–1.0)	624
Wang (2021)	COVID‐19 will be under control in the coming month	AOR: 1.03 (0.91–1.18)	15428
Zewude et al. (2021)	Belief that COVID exists in the country Belief that COVID causes severe illness Think can be infected by covid	AOR: 1.60 (0.67–3.84) AOR: 0.71 (0.35–1.42) AOR: 0.68 (0.35–1.32)	379
Van den Broek Altenburg (2021)	Motivated to adhere to handwashing guidelines by Protect family Protect public Conformity Family/friends recommend Physician recommendation Politician recommendation Legal restrictions Self‐protection	Multiple regression coefficients: 0.516 0.207 −0.139 −0.176 0.107 0.135 0.075 0.24	4311
Norman (2020)	Instrumental attitude	*r* = 0.3	477
Kowalski (2020a) Study 1	Motivation for adherence: internal, e.g., to protect my and/or others health	*r *= 0.22	840
Stojanovic (2021)	Health concerns about oneself Health concerns about others	Multiple regression coefficients: −0.333 (−0.703 to 0.036) 0.831 (0.376 to 1.286)	1332
Dixon et al. (2022)	Your COVID‐19 symptoms will last a long time You could get COVID‐19 again beliefs about Belief about cause (my not wearing a face covering) Belief about cause (other people not keeping their distance)	Multiple regression coefficients: 0.027 0.064 0.028 0.074	2969

Abbreviations: AOR, adjusted odds ratio; CI, confidence interval; OR, odds ratio.

* *p* < 0.05.

## 7 DISCUSSION

### 7.1 Summary of main results

This systematic review aimed to synthesise the evidence examining psychosocial factors that determine the uptake and adherence to handwashing and hand sanitising behaviours for reducing the risk of infection or transmission of severe acute respiratory coronavirus 2 (SARS‐CoV‐2) in the general public.

The review forms part of the CoHeRe project (Hanratty et al., 2022). This interdisciplinary, multinational project has involved the development of an Evidence and Gap Map to identify and summarise current research on determinants of COVID‐19 protective behaviours, and a series of individual reviews examining the determinants of these specific behaviours (Hanratty et al., 2022).

This review provides one of the first studies to synthesis, using meta‐analyses and narrative summaries, evidence on the malleable factors that are most associated with handwashing and hand sanitising behaviours. The focus on only malleable factors, excluding determinants such as demographic characteristics, is important, as it provides evidence to inform the development of interventions promoting handwashing. Specifically, intervention targeted at malleable determinants of protective behaviours could be used as part of effective public health messages implemented to promote handwashing and hand sanitising behaviours in the context of potential future waves of COVID‐19, and other respiratory infections with pandemic potential.

A total of 56 studies were suitable for inclusion in the review, representing 199,376 participants. All the included studies were online, cross‐sectional studies, with the majority being published in the United States (*n* = 12) or China (*n* = 10). Thirty‐five studies were published in 2021, within the first 12 months of the COVID‐19 pandemic being declared. Across all 56 included studies the most common malleable determinants of handwashing behaviours were perceived susceptibility [*n* = 25 studies (45%) and perceived severity (*n* = 21 studies (38%)]. Smaller numbers of studies examined determinants such as COVID‐related anxiety and perceived effectiveness of handwashing [*n* = 3 (5%) and 3 (5%) respectively].

Overall findings based on the results of the meta‐analysis indicated that emotions about COVID, knowledge of COVID‐19, and perceived social norms regarding behaviours were among the malleable determinants most associated with handwashing. Perceived effectiveness, attitudes towards behaviours, and self‐efficacy were also linked with these behaviours, albeit with a smaller effect.

Perceived severity and susceptibility of COVID‐19 were not associated with handwashing behaviour.

Findings from the meta‐analysis and narrative synthesis did therefore show some agreement, particularly related to the association between handwashing behaviours and people's emotions around COVID‐19.

It is important to note that the meta‐analyses presented in this review have a high degree of heterogeneity (apart from the two meta‐analyses that found no significant association between handwashing and perceived severity and susceptibility). This heterogeneity could be a result of variation in the measurement or operational definition of the determinants, or variation in the measurement or operational definition of handwashing, or variation in the timing of the study in relation to government‐led initiatives or mandates within each country. Furthermore, the evidence presented in the review is drawn from cross‐sectional studies, which prevents any conclusions being drawn that go beyond associations between variables. In other words, the review does not help us to understand how change in the determinants might be related to change in handwashing behaviour. This is a gap for further research.

### 7.2 Overall completeness and applicability of evidence

To the best of our knowledge, the evidence presented in this review represents the entirety of research to date (completed searches October 2021) on malleable determinants of handwashing as a COVID‐related behaviour. During this review, we followed a pre‐registered peer‐reviewed protocol that was developed in consultation with expert stakeholders and methods experts. A comprehensive search was conducted to identify relevant studies and a team of experts and reviewers worked independently to select studies using the predetermined eligibility criteria and extract outcome data using a standardised data extraction form.

Twenty‐eight studies (61,956 participants) were suitable for pooling of data in the meta‐analysis.

Samples from 22 countries were represented in the 56 included studies. The majority of these being from the USA (*n* = 12) and China (*n* = 10). Given that COVID‐19 is a global pandemic the more narrow geographical coverage of the studies may limit the applicability of the evidence.

This was a large review examining data from a total of 199,376 participants across the 56 studies on one COVID‐related behaviour. The research on COVID‐19 has been published at a rapid rate since the beginning of the pandemic. A rapid review conducted in 2020 as part of the CoHeRe project (Hanratty et al., 2021), included 54 studies looking at 9 different COVID‐related behaviours. This review included 56 studies looking at handwashing alone, evidencing the rapidly increasing volume of COVID‐related research. This review and subsequent reviews are highly applicable to those involved in the development and implementation of public health decisions, interventions, and messaging to promote health behaviours in the context of COVID‐19, and other respiratory infections.

### 7.3 Quality of the evidence

The majority of the included studies were of fair methodological quality. However, a number of studies [*n* = 8 (15%)], were assessed as being of low quality due to the presence of methodological limitations, primarily, lack of clarity over recruitment and methods (see Table 4).

### 7.4 Potential biases in the review process

To limit potential bias, a systematic approach, which included input from an information retrieval specialist, was used to plan and conduct the searches and the study identification process. Searches also included information sources such as trial registers and repositories, which were used to identify recent and rapidly emerging evidence. Other strengths include the extensive use of stakeholder involvement via advisory panel input, and through participation of the Cochrane Crowd, who contributed to the screening of a large number of potential records for inclusion. Screening was completed by three reviewers independently. In addition, 20% of all studies were checked by a second author throughout the screening and extraction process.

### 7.5 Agreements and disagreements with other studies or reviews

There are a number of related published and ongoing reviews on determinants of COVID‐19 health‐related behaviours but none with the broad scope of this review. A recently published review by (Liang et al., 2022) examined the psychosocial determinant of hand hygiene, mask wearing and physical distancing. They included 24 studies examining hand hygiene and applied the Risk, Attitudes, Norms, Abilities, and Self‐Regulation (RANAS) model when determining determinants of interest. They found that perceived susceptibility and severity was not a significant determinant of hand hygiene, while knowledge, perceived norms, and self‐efficacy was significant. Our findings concur and add to those found by (Liang et al., 2022).

## 8 AUTHORS’ CONCLUSIONS

### 8.1 Implications for practice and policy

The findings from this review indicate that emotions towards COVID (COVID‐related anxiety and worry), knowledge about COVID and perceived social norms are the determinants most associated with handwashing. While determinants like perceived severity and perceived susceptibility have little to no effect on handwashing behaviour. An understanding of how these malleable determinants impact hand washing behaviour provides evidence to inform the development of future interventions, and public health campaigns. Moreover, this evidence provides important insights regarding the determinants of handwashing for potential future waves of COVID‐19, and other respiratory infections.

### 8.2 Implications for research

The volume of research on COVID has rapidly increased from the beginning of the pandemic, and continues to emerge. Increased demand to understand the determinants of COVID‐19 related behaviour has resulted studies being completed rapidly, often at the expense of the quality of the research (Park et al., 2021). Other studies have similarly pointed to the need for well‐designed, good quality studies (Park et al., 2021), on the determinants of COVID related behaviour. In addition, the majority of our studies were from high‐income countries, largely the USA and China. COVID‐19 is a global pandemic, thus we need to understand how and if the determinants of behaviour vary globally. Finally, the most commonly reported determinants were perceived susceptibility and severity. Our research has shown these to have little to no effect on handwashing, albeit these results must be interpreted cautiously. Determinants such as emotions relating to COVID, knowledge about COVID and social norms were less commonly reported; however had a larger effect on handwashing behaviour. These determinants should be considered further.

## CONTRIBUTIONS OF AUTHORS

This review was undertaken by a team with substantial expertise in systematic reviews, health behaviour and infectious diseases. Professor Martin Dempster, Principal Investigator (PI) of the project had overall responsibility for its conduct and delivery. Dr Sean O'Connor, Dr Rachel Leonard and Dr Jennifer Hanratty was responsible for the day‐to‐day operation of the review, led screening, data extraction, quality assessment and reporting. Dr Ciara Keenan acted as an information retrieval specialist, designed and conducted the searches, and contributed to screening and data extraction. Ariana Axiaq, Yuan Chi, Victoria Hawkins, Kerry Campbell, Ceri Welsh, Anna Volz and Janet Ferguson contributed to screening and data‐extraction. Professor Miller acted as advisor on evidence synthesis methodology. Dr Bradley was the content expert on communicable diseases and reviewed and commented on drafts.

Dr Jennifer Hanratty is a psychologist and expert in evidence synthesis. She has worked in evidence synthesis since 2012 and published reviews with Campbell, Cochrane and NIHR Health Technology Assessment among others, was editor with Campbell Education Co‐ordinating group, Fellow with Campbell UK & Ireland and an invited member of the international advisory board for Evidence Synthesis Ireland.

Dr Sean O'Connor is a Physiotherapist and an experienced health care researcher. He has undertaken a number of systematic reviews and studies related to behavioural interventions, including in the context of COVID‐19. He has an extensive knowledge of theory‐based implementation models for maximising integration of evidence into practice, systematic review methods including methodological quality/risk of bias assessment and the examination of stakeholder perspectives in healthcare delivery.

Dr Rachel Leonard is a Social Worker and an experienced health care researcher. She has experience of conducting and leading on a number of systematic reviews, meta‐analyses, and studies related to health interventions.

Dr Ciara Keenan is a methods editor and information retrieval specialist for the Campbell collaboration. She has considerable experience conducting and leading the creation of EGMs and systematic reviews.

Ariana Axiaq is a third year medical student with an interest in health promotion and extensive research experience in thematic analyses and systematic reviews.

Yuan Chi is Cochrane Information Specialist, Founder CEO of Yealth Technology, core team member with Cochrane COVID‐19 Study Register (https://covid-19.cochrane.org/), and the only Chinese executive team member for COVID19 Recommendation Map. She has 8 years experience on evidence synthesis, assisted 50+ international projects, resulting in 11 authorships and 28 recommendations from Cochrane editors, information specialists and authors.

Dr Janet Ferguson is a researcher with a background in the use of technology to provide training in social communication to parents of autistic children. She has experience in assisting and conducting Systematic Literature Reviews across a number of related topics.

Victoria Hawkins is a Masters student in the School of Psyhcology, QUB.

Kerry Campbell is a PhD student in the School of Psyhcology, QUB.

Ceri Welsh is a PhD student in the School of Psyhcology, QUB.

Anna Volz is a third year undergraduate psychology student.

Professor Sarah Miller is Director of Campbell UK & Ireland. She is co‐chair and co‐editor of the Campbell Education Coordinating Group.

Dr Bradley is a consultant in public health medicine and clinical lecturer in public health and was consultant in health protection (communicable disease control) before taking up his current post. His publishing record includes several systematic reviews and studies of healthcare‐related behaviour. He is a member of the Northern Ireland COVID‐19 Modelling and Behaviour Change Groups.

Professor Dempster, is a registered Health Psychologist and Chartered Statistician, with over 20 years’ experience in conducting research on the determinants of behaviour change. He has published 14 reviews, including reviews of effectiveness and reviews of covariates. He is currently a member of the Northern Ireland Public Health Agency COVID‐19 Behaviour Change Group.

- Content: Bradley, Dempster, Hanratty, Miller, Keenan, O'Connor, Leonard
- Systematic review methods: Hanratty, Miller, Dempster, Keenan, O'Connor, Leonard
- Statistical analysis: Dempster, Miller, Hanratty, Keenan, O'Connor, Leonard, Ferguson, Axiaq, Chi, Volz, Welsh, Campbell, Hawkins
- Qualitative Evidence Synthesis: n/a
- Information retrieval: Hanratty, Keenan, O'Connor

## DECLARATIONS OF INTEREST

None of the review team have any present or past affiliations or other involvement in any organisation or entity with an interest in the review's findings that might lead to a real or perceived conflict of interest.

## PLANS FOR UPDATING THIS REVIEW

This review will not be updated by the project team as the end of our funding period is on 18th October 2022. Teams interested in building on the review or contributing to updating beyond the project end date are encouraged to contact the corresponding author.

## SOURCES OF SUPPORT

### Internal sources

- No sources of support provided

### External sources

- No sources of support provided

## Supporting information

Supporting information.
